# Supplementary figures and images for: Transmissible cancers and the evolution of sex under the Red Queen hypothesis
Source: PLoS Biol. 2020 Nov 19;18(11):e3000916. doi: 10.1371/journal.pbio.3000916 (PMC7676742; doi:10.1371/journal.pbio.3000916)

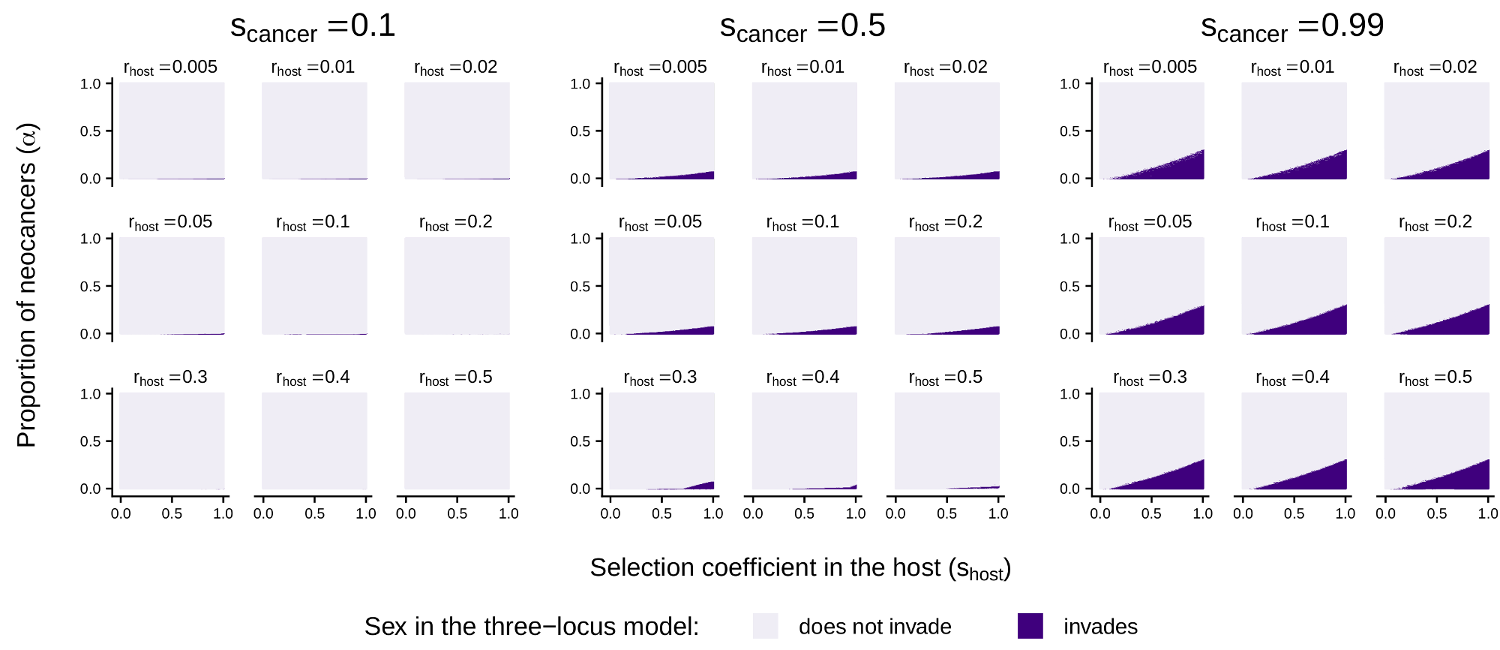

Supplement: S1 Fig — Sensitivity of the three-locus population genetic model to the selection coefficients (shost,scancer) and to the proportion of neocancers that are recently derived from the original host (α). The conditions under which sex can invade are more restricted when sex associates with a high recombination rate (leading to a high recombination load). Sex without genetic mixing is neutral compared to asexual reproduction (i.e., if rhost = 0 in our haploid case, not shown). Therefore, sex is more strongly favoured if it associates with an intermediate recombination rate rhost. See Fig 1 for more details. (TIF) [file pbio.3000916.s001.tif]

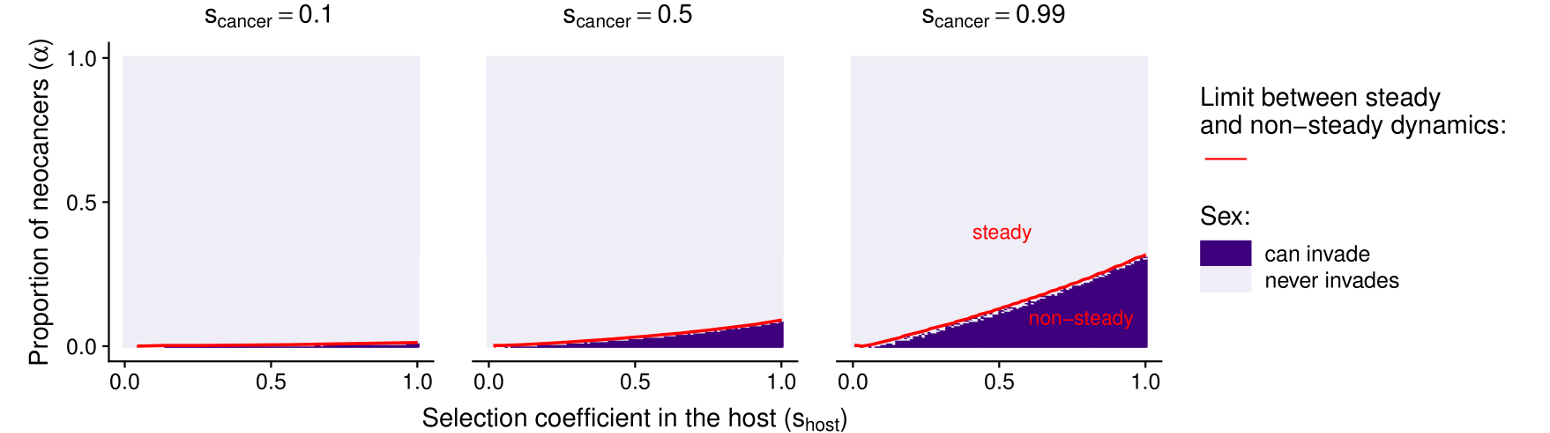

Supplement: S2 Fig — Sensitivity of the population genetic models to the selection coefficients (shost,scancer) and to the proportion of transmissible neocancers that are recently derived from the original host (α). Red lines delimit the parameter spaces leading to non-steady and steady coevolutionary dynamics. The dynamic is defined as ‘steady’ when the variance in genotypic frequencies over 500 time steps is below 10−10. Dark purple indicates conditions under which a modifier allele associated with obligate sexual reproduction (and with recombination, at least for one of the recombination rates tested) can invade in at least one of the 100 simulation runs. We get the same results as in Fig 1. (TIF) [file pbio.3000916.s002.tif]

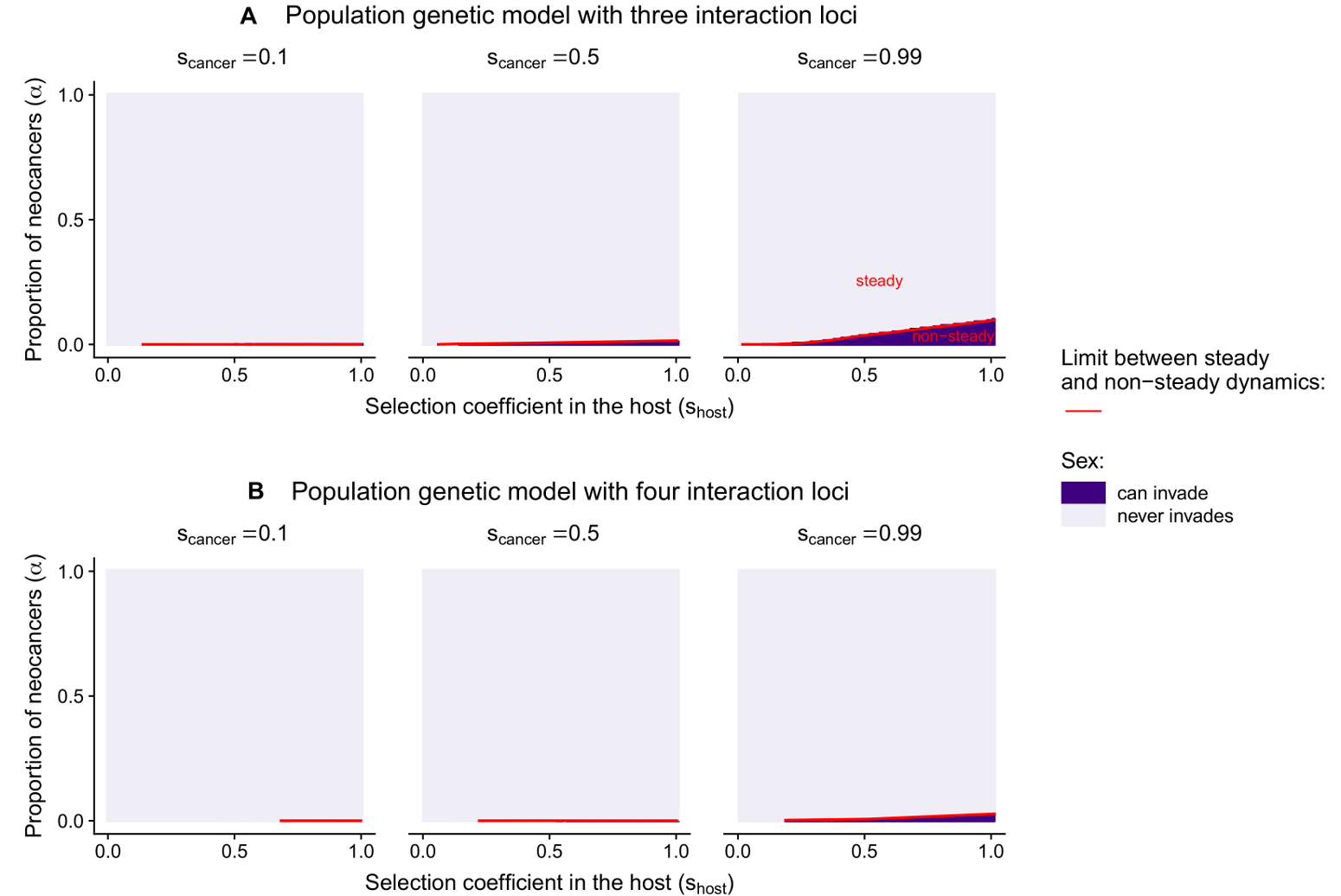

Supplement: S3 Fig — Sensitivity of the population genetic models to the selection coefficients (shost,scancer) and to the proportion of transmissible neocancers that are recently derived from the original host (α). Red lines delimit the parameter spaces leading to non-steady and steady coevolutionary dynamics. The dynamic is defined as ‘steady’ when the variance in genotypic frequencies over 500 time steps is below 10−10. Dark purple indicates conditions under which a modifier allele associated with sexual reproduction (and with recombination, at least for one of the recombination rates tested) can invade in at least one of the 100 simulation runs. Sex is favoured mostly within a restricted genotypic space under the Red Queen hypothesis (as shown in [38]; but see [84] and [85] accounting for other evolutionary processes favouring sexual reproduction). Additionally, neoplasia dampens coevolutionary cycling even when considering more than two interaction loci. (TIF) [file pbio.3000916.s003.tif]

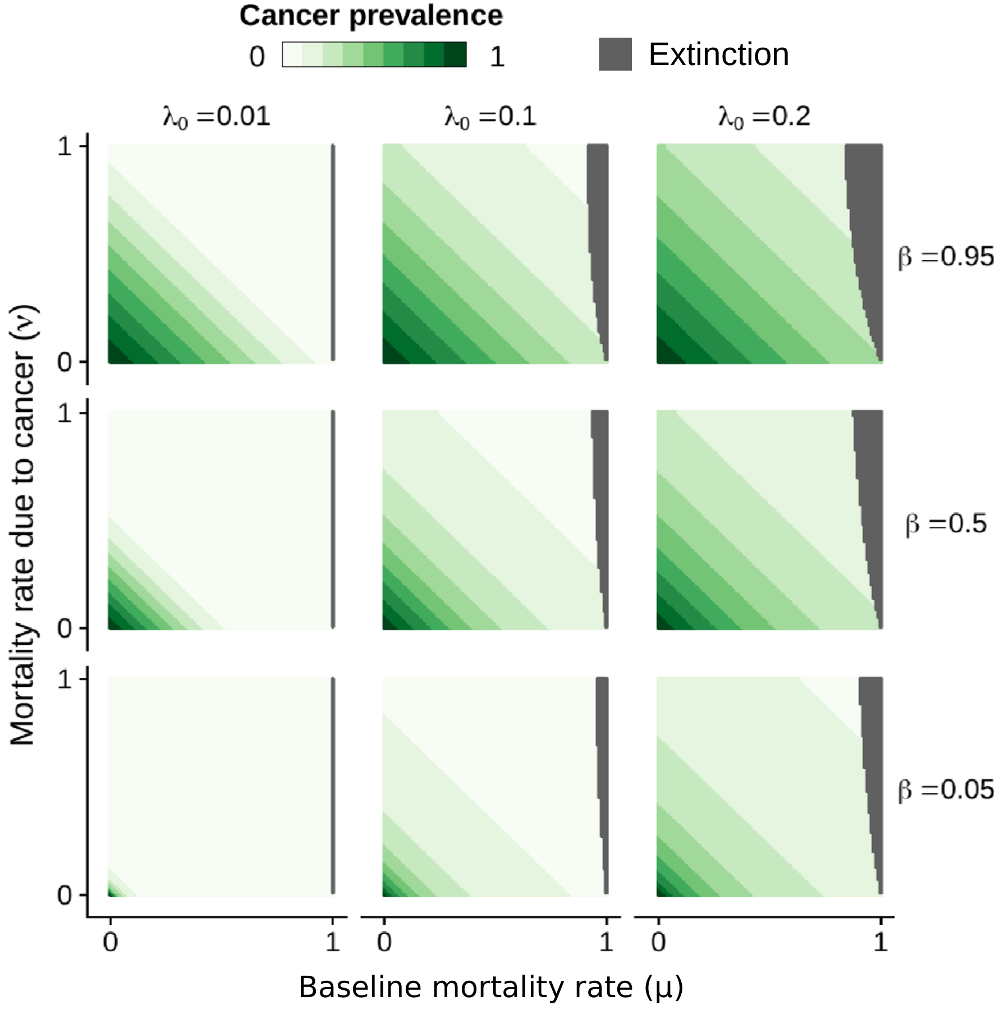

Supplement: S4 Fig — A high prevalence associates with a high selection coefficient in the host (high s^host, Fig 4B). In grey, we represent the conditions under which the host population gets extinct, assuming that the baseline birth rate b equals to one (condition leading to extinction: μ+νP*(λ0,β,μ,ν)>b; see S3 Appendix). (TIF) [file pbio.3000916.s004.tif]

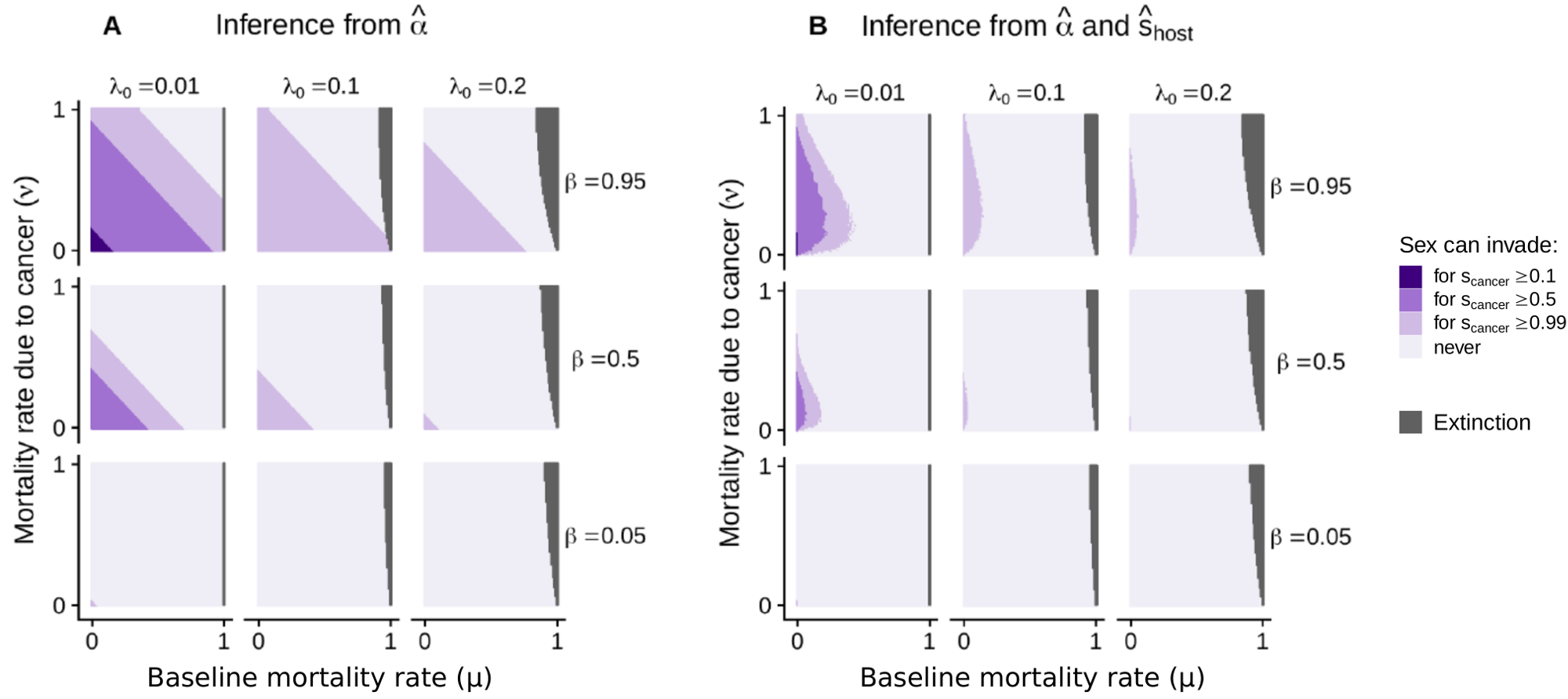

Supplement: S5 Fig — At equilibrium, we determine the values of (α^,s^host), and the conditions favouring the evolution of sex are inferred (A) from α^ only or (B) from (α^,s^host). Decreased rate of neoplasia (λ0) leads to the conditions of α^ that are prone to the evolution of sex (A). Nonetheless, it also associates with a decrease in the selection coefficient caused by transmissible cancers (Fig 4B), thereby inhibiting the evolution of sex (B). In grey, we represent the conditions under which the host population gets extinct, assuming that the baseline birth rate b equals to one (condition leading to extinction: μ+νP*(λ0,β,μ,ν)>b; see S3 Appendix). See Fig 1 for more details. (TIF) [file pbio.3000916.s005.tif]

$s_{\text{cancer}} = 0.1$  $s_{\text{cancer}} = 0.5$  $s_{\text{cancer}} = 0.99$ 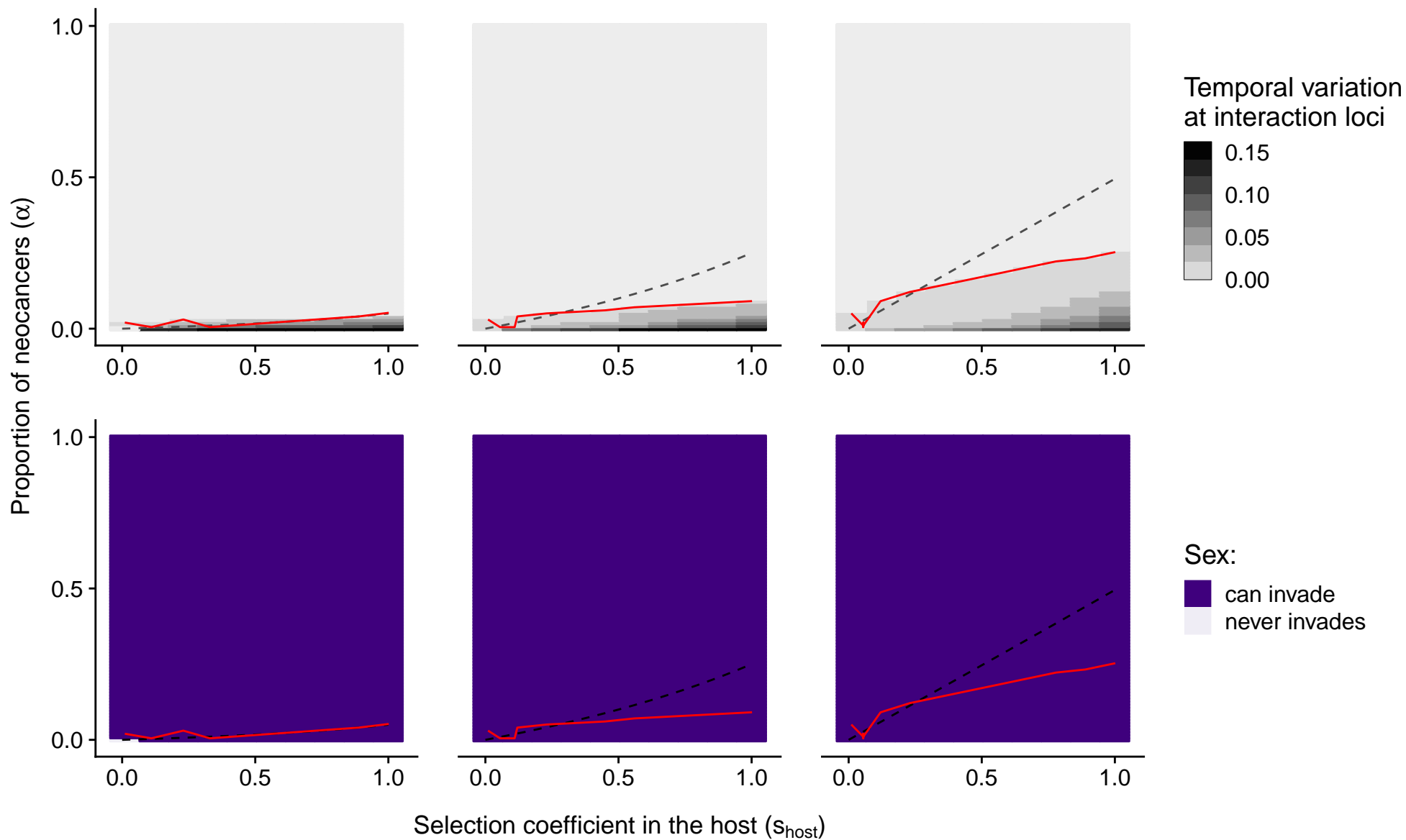

Supplement: S1 Source Code — (ZIP) [file pbio.3000916.s010.zip › SimulationCode/Graphes/CombinPlot1.pdf]

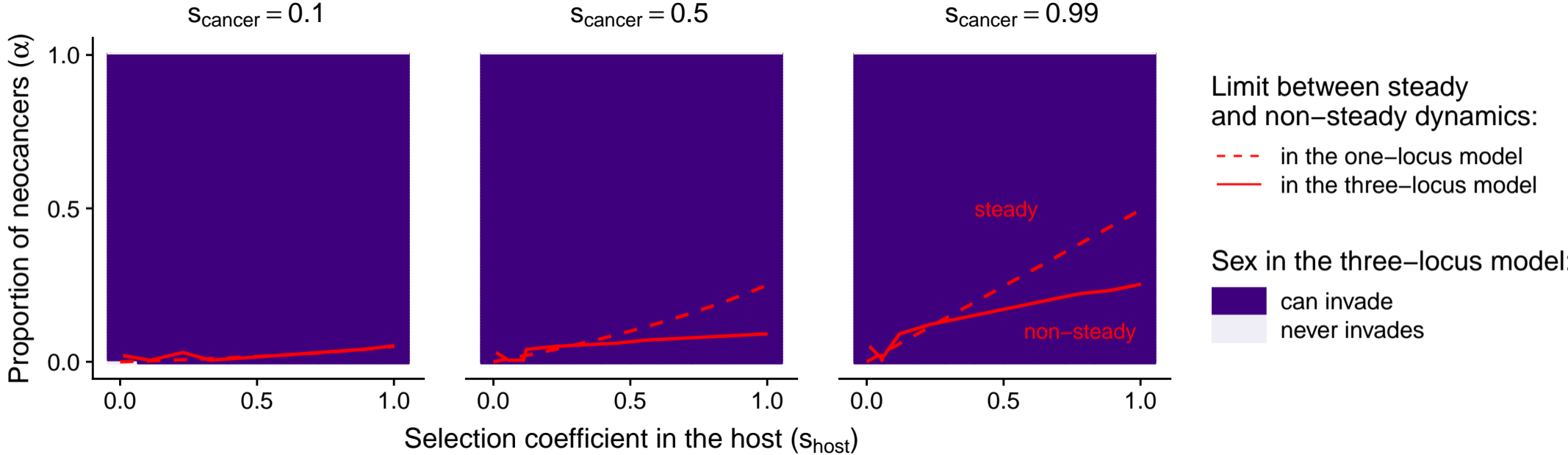

Supplement: S1 Source Code — (ZIP) [file pbio.3000916.s010.zip › SimulationCode/Graphes/CombinPlot2.pdf]

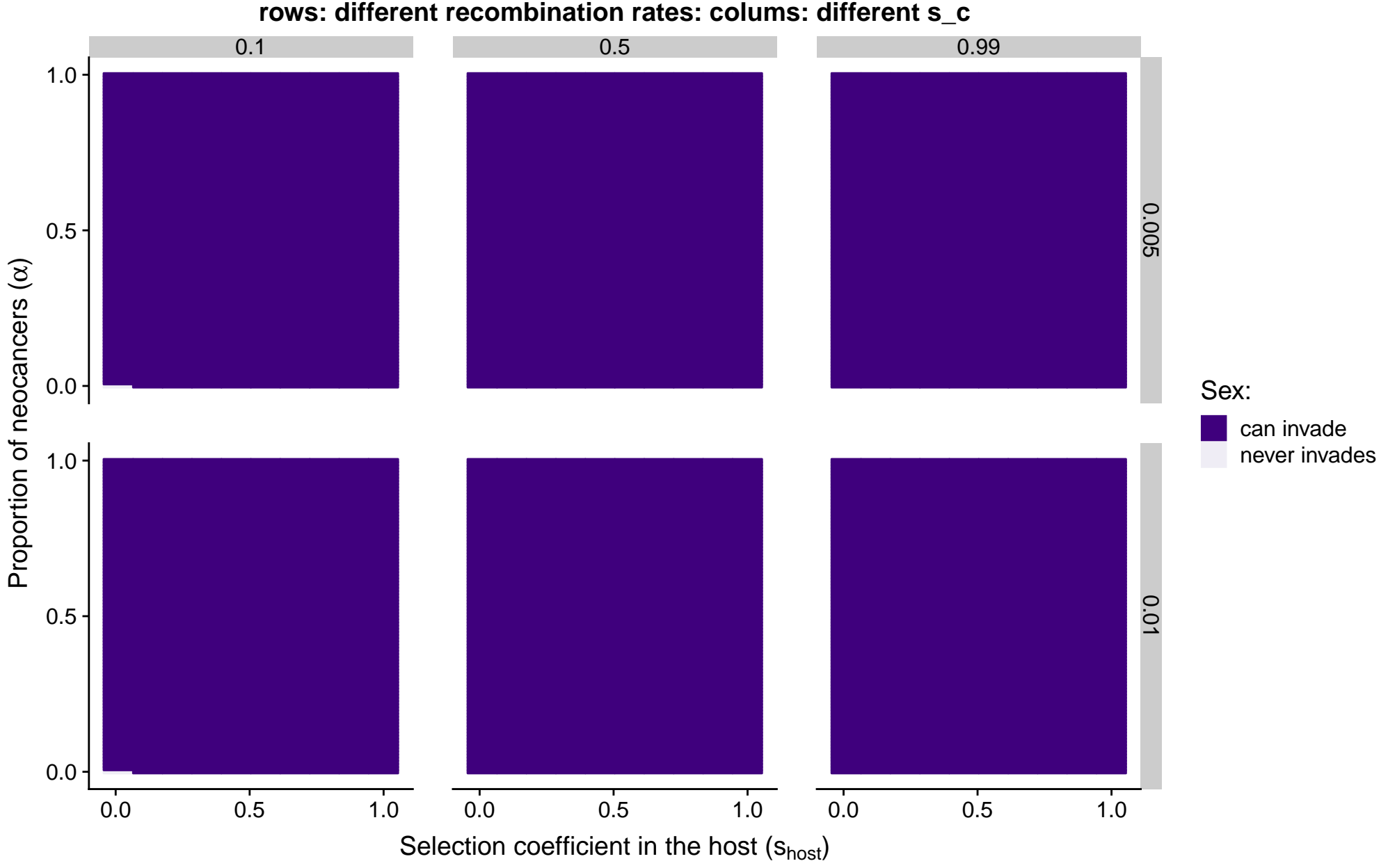

Supplement: S1 Source Code — (ZIP) [file pbio.3000916.s010.zip › SimulationCode/Graphes/CombinPlot3.pdf]

Frequency

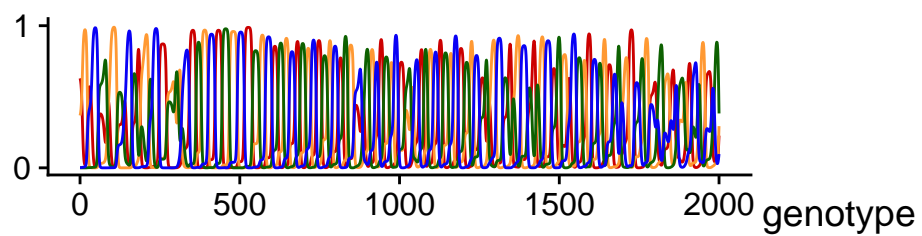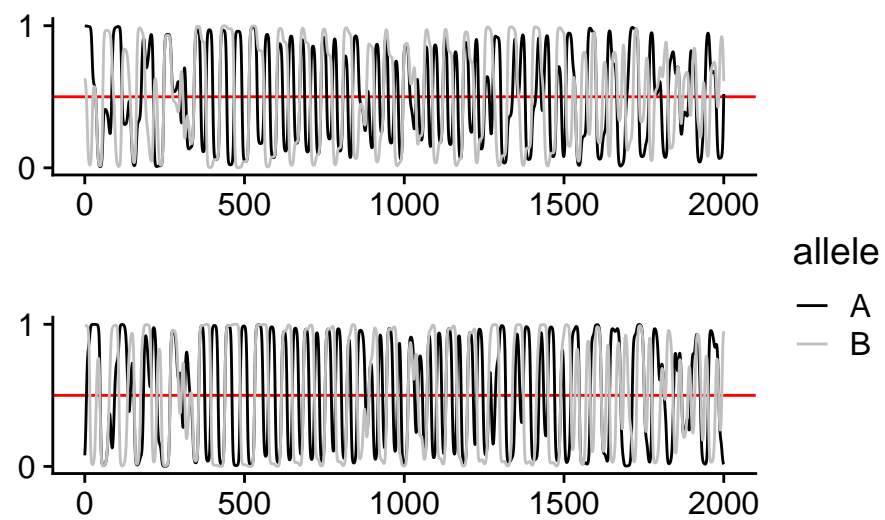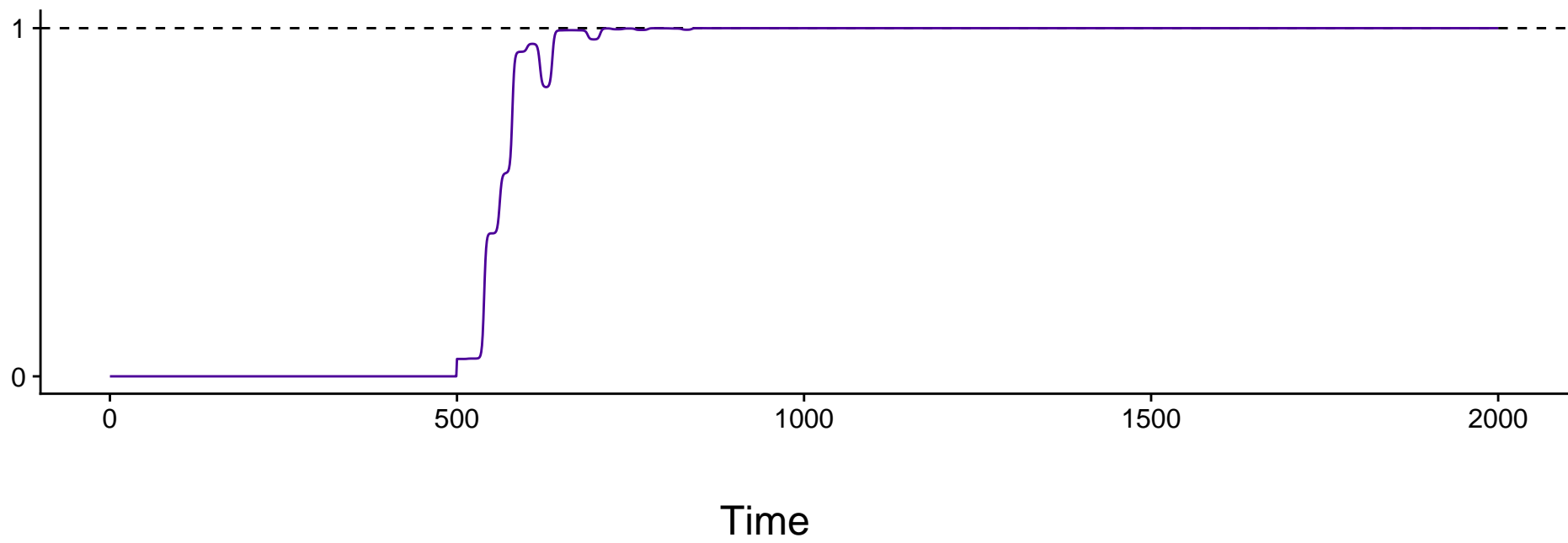

Supplement: S1 Source Code — (ZIP) [file pbio.3000916.s010.zip › SimulationCode/Graphes/SingleSimulation.pdf]

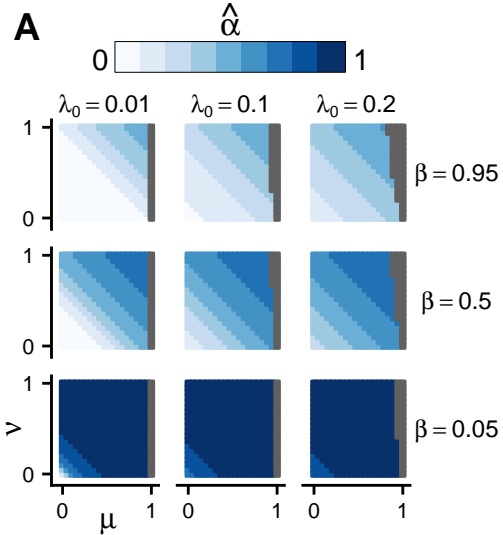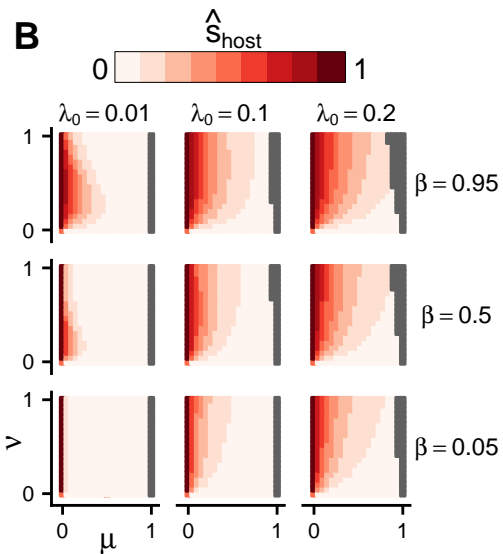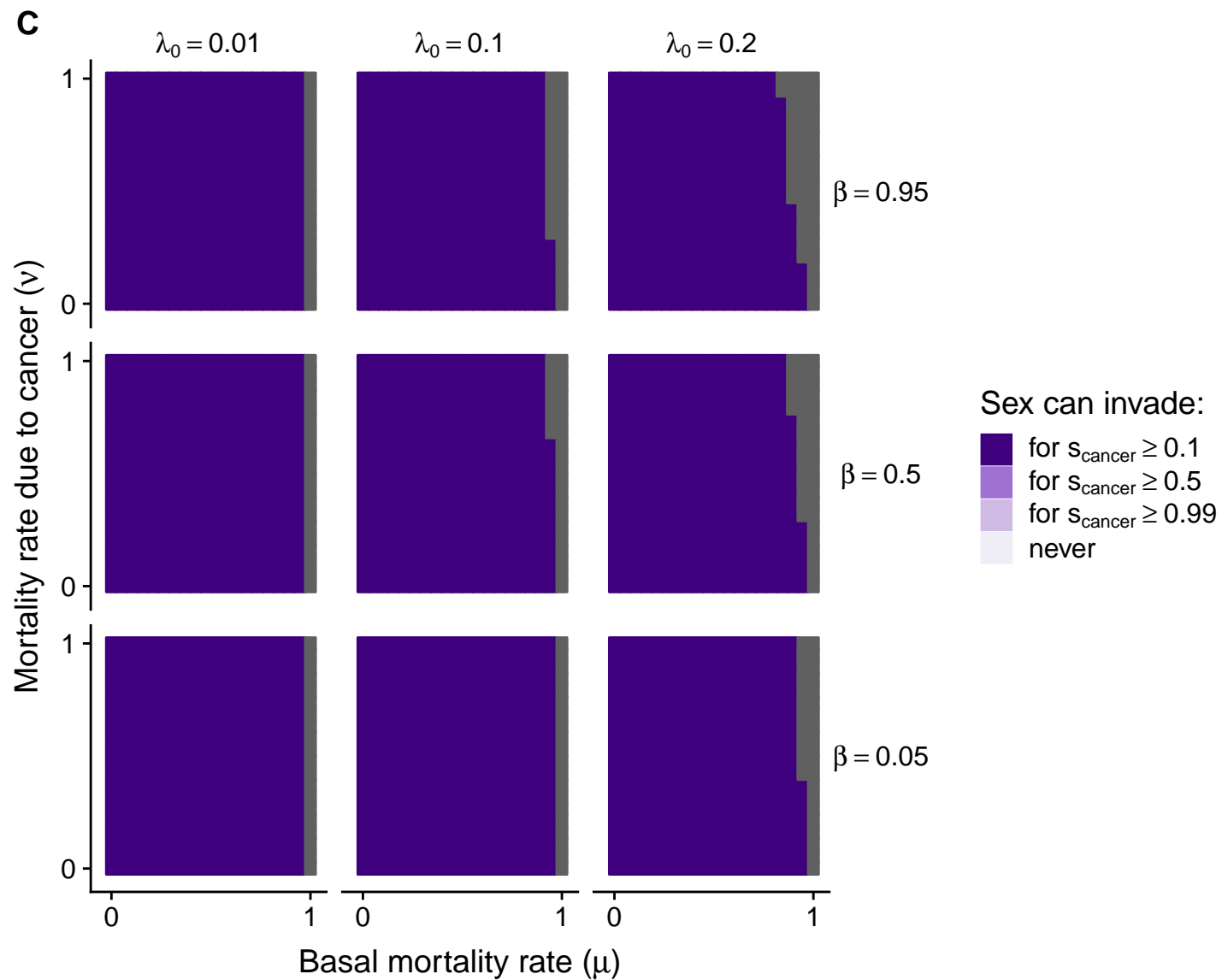

Supplement: S1 Source Code — (ZIP) [file pbio.3000916.s010.zip › SimulationCode/Graphes/plotDemography.pdf]

# Cancer prevalence

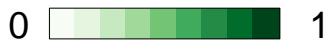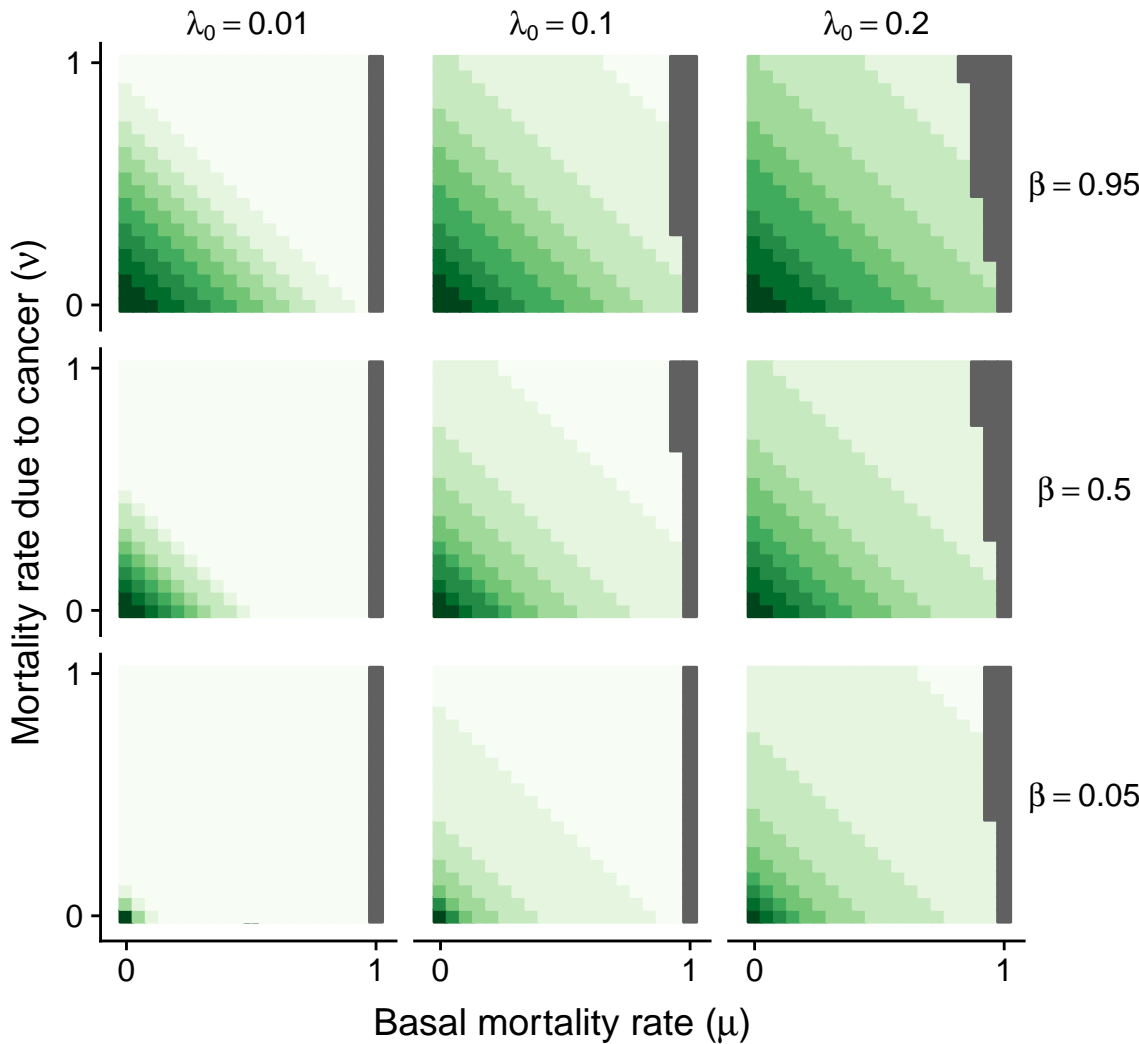

Supplement: S1 Source Code — (ZIP) [file pbio.3000916.s010.zip › SimulationCode/Graphes/plotDemographyPrevalence.pdf]
