# Supplementary material for: Transmissible cancers and the evolution of sex under the Red Queen hypothesis
Source: PLoS Biol. 2020 Nov 19;18(11):e3000916. doi: 10.1371/journal.pbio.3000916 (PMC7676742; doi:10.1371/journal.pbio.3000916)
Supplement: S1 Appendix — (PDF) [file pbio.3000916.s006.pdf]

S1 APPENDIX: Analytical derivation of a simplified one-locus population genetic model

Purpose

To gain further insights into the phenomenon presented in the main manuscript, we consider a simplified one-locus population genetic model and solve it analytically.

Hosts and transmissible cancers are haploid. This simplified population genetic model is based on a single autosomal haploid locus A with two possible alleles (A/a), controlling the interaction between hosts and transmissible cancers (with selection coefficients s_{host} in case of a match, and s_{cancer} in case of a mismatch, respectively). Time is discrete, and we assume that a proportion of transmissible cancers α derives from neocancers.

Contrary to the population genetic model analyzed numerically in the manuscript, this model does not include a modifier locus controlling the reproduction mode of the host. Here, our aim is to investigate analytically the conditions under which coevolutionary cycling (so-called 'Red Queen dynamics') is taking place between hosts and transmissible cancers. Likewise, we assume there is no mutation.

Below, we show that all equilibrium points are unstable for:

$$\boxed{\alpha < \frac{s_{\text{host}}\, s_{\text{cancer}}}{(2 - s_{\text{host}})(2 - s_{\text{cancer}}) + s_{\text{host}}\, s_{\text{cancer}}}} \tag{1}$$

Under this condition, the long-term behaviour of the system is not steady. We cannot show analytically that this corresponds to a limit cycle; it could also be chaotic dynamics. In both cases, such non-steady long-term behaviour gives scope for the evolution of sex under the Red Queen Hypothesis.

Recursive equations

The systems of equations describing the changes in frequency of allele A in the host and the transmissible cancers (f_{h} and f_{c}) over one generation are:

$$\begin{cases} f'_{\text{h}} &= \dfrac{f_{\text{h}}\, [\,(1 - s_{\text{host}})\; f^*_{\text{c}} + (1 - f^*_{\text{c}})\,]}{f_{\text{h}}\, [\,(1 - s_{\text{host}})\; f^*_{\text{c}} + (1 - f^*_{\text{c}})\,] + (1 - f_{\text{h}})\; [\,f^*_{\text{c}} + (1 - s_{\text{host}})\; (1 - f^*_{\text{c}})\,]} \\ f'_{\text{c}} &= \dfrac{f^*_{\text{c}}\; [\,f_{\text{h}} + (1 - s_{\text{cancer}})\; (1 - f_{\text{h}})\,]}{f^*_{\text{c}}\; [\,f_{\text{h}} + (1 - s_{\text{cancer}})\; (1 - f_{\text{h}})\,] + (1 - f^*_{\text{c}})\; [\,(1 - s_{\text{cancer}})\; f_{\text{h}} + (1 - f_{\text{h}})\,]} \end{cases} \tag{2}$$

With

$$f^*_{\text{c}} = \alpha\, f_{\text{h}} + (1 - \alpha)\; f_{\text{c}} \tag{3}$$

Parameter α corresponds to the proportion of transmissible cancers that derive from neocancers. Hosts suffer a fitness cost if they get infected by cancers with the same genotype than their own (fitness coefficient $1 - s_{\text{host}}$). Transmissible cancers suffer a fitness cost if they infect hosts with a different genotype than their own (fitness coefficient $1 - s_{\text{cancer}}$).

This system of equations can be simplified as:

$$\begin{cases} f'_{\text{h}} &= \dfrac{f_{\text{h}}\; [\,1 - s_{\text{host}}\, f^*_{\text{c}}\,]}{1 + s_{\text{host}}\, [f_{\text{h}} + f^*_{\text{c}} - 1 - 2\, f_{\text{h}}\, f^*_{\text{c}}]} \\ f'_{\text{c}} &= \dfrac{f^*_{\text{c}}\; [\,1 - s_{\text{cancer}}\; (1 - f_{\text{h}})\,]}{1 + s_{\text{cancer}}\, [2\, f_{\text{h}}\, f^*_{\text{c}} - f_{\text{h}} - f^*_{\text{c}}]} \end{cases} \tag{4}$$

With $0 \leq \alpha \leq 1$, $0 < s_{\text{host}} < 1$ and $0 < s_{\text{cancer}} < 1$.

Equilibria

This system of equations is characterized by three or five equilibrium points depending on parameter α:

- Fixation of different alleles in host and cancer: $(f_\mathrm{h}, f_\mathrm{c}) = (1,0)$ or $(0,1)$ **only if** $\boldsymbol{\alpha = 0}$
- Fixation of the same allele in host and cancer: $(f_\mathrm{h}, f_\mathrm{c}) = (0,0)$ or $(1,1)$
- Equal frequencies of the two alleles in both host and cancer: $(f_\mathrm{h}, f_\mathrm{c}) = (0.5, 0.5)$

Local stability

At each equilibrium point, the local stability can be inferred from the eigenvalues of the Jacobian matrix $\mathbf{J}$. The complete expression of $\mathbf{J}$ is very unwieldy. Below, we only show the expressions of the Jacobian matrices evaluated at the equilibrium points.

1. Fixation of different alleles (if $\alpha = 0$)

Around the equilibrium points $(0,1)$ and $(1,0)$, the expression of the Jacobian matrix is:

$$\mathbf{J}_{(0,1),(1,0)|\alpha=0} = \begin{bmatrix} 1 - s_\mathrm{host} & 0 \\ 0 & \frac{1}{1-s_\mathrm{cancer}} \end{bmatrix} \tag{5}$$

Given that the eigenvalue $\frac{1}{1-s_\mathrm{cancer}} > 1$ (on the diagonal), those equilibrium points are always locally **unstable**.

2. Fixation of the same allele

Around the equilibrium points $(0,0)$ and $(1,1)$, the expression of the Jacobian matrix is:

$$\mathbf{J}_{(0,0),(1,1)} = \begin{bmatrix} \frac{1}{1-s_\mathrm{host}} & 0 \\ \alpha\,(1 - s_\mathrm{cancer}) & (1 - s_\mathrm{cancer})(1-\alpha) \end{bmatrix} \tag{6}$$

Given that the eigenvalue $\frac{1}{1-s_\mathrm{host}} > 1$ (on the diagonal), those equilibrium points are always locally **unstable**.

3. Equal frequencies of the two alleles

Around the equilibrium points $(0.5, 0.5)$, the expression of the Jacobian matrix is:

$$\mathbf{J}^* = \begin{bmatrix} 1 - \frac{\alpha\, s_\mathrm{host}}{2-s_\mathrm{host}} & \frac{-s_\mathrm{host}\,(1-\alpha)}{2-s_\mathrm{host}} \\ \alpha + \frac{s_\mathrm{cancer}}{2-s_\mathrm{cancer}} & 1-\alpha \end{bmatrix} \tag{7}$$

We can show that this equilibrium point is unstable for:

$$\alpha < \frac{s_\mathrm{host}\, s_\mathrm{cancer}}{(2 - s_\mathrm{host})(2 - s_\mathrm{cancer}) + s_\mathrm{host}\, s_\mathrm{cancer}} \tag{8}$$

Proof:
The trace and determinant of $\mathbf{J}^*$ are:

$$\mathrm{trace}\,(\mathbf{J}^*) = 2 - \alpha - \frac{\alpha\, s_\mathrm{host}}{2 - s_\mathrm{host}} \tag{9}$$

$$\det\,(\mathbf{J}^*) = \frac{(1-\alpha)\ \left[(2 - s_\mathrm{cancer})\,(2 - s_\mathrm{host}\,(1+\alpha)) + s_\mathrm{host}\,(\alpha\,(2 - s_\mathrm{cancer}) + s_\mathrm{cancer})\right]}{(2 - s_\mathrm{host})(2 - s_\mathrm{cancer})} \tag{10}$$

The eigenvalues can be expressed as:

$$\lambda = \frac{\mathrm{trace}\,(\mathbf{J}^*) \pm \sqrt{\mathrm{trace}\,(\mathbf{J}^*)^2 - 4\det\,(\mathbf{J}^*)}}{2} \tag{11}$$

The eigenvalues are either complex or real, depending on the sign of:

$$\mathrm{trace}\left(\mathbf{J}^*\right)^2 - 4\det\left(\mathbf{J}^*\right) = \frac{4\left[\left(2-s_{\mathrm{cancer}}\right)\alpha^2 - 2\,s_{\mathrm{host}}\left(1-s_{\mathrm{cancer}}\right)\left(2-s_{\mathrm{host}}\right)\alpha - s_{\mathrm{host}}\,s_{\mathrm{cancer}}\left(2-s_{\mathrm{host}}\right)\right]}{(s_{\mathrm{host}}-2)^2(2-s_{\mathrm{cancer}})} \tag{12}$$

which has the same sign as the polynomial function:

$$P(\alpha) = \left(2-s_{\mathrm{cancer}}\right)\alpha^2 - 2\,s_{\mathrm{host}}\left(1-s_{\mathrm{cancer}}\right)\left(2-s_{\mathrm{host}}\right)\alpha - s_{\mathrm{host}}\,s_{\mathrm{cancer}}\left(2-s_{\mathrm{host}}\right) \tag{13}$$

The discriminant of this polynomial function is positive:

$$\Delta_P = 4\,s_{\mathrm{host}}^2\left(1-s_{\mathrm{cancer}}\right)^2\left(2-s_{\mathrm{host}}\right)^2 + 4\,\left(2-s_{\mathrm{cancer}}\right)\left(2-s_{\mathrm{host}}\right)s_{\mathrm{host}}\,s_{\mathrm{cancer}} \tag{14}$$

The product of the roots of this polynomial function is negative (Vieta's formula). Therefore, this polynomial function is characterized by a single positive root A_+:

$$A_+ = \frac{2\,s_{\mathrm{host}}\left(1-s_{\mathrm{cancer}}\right)\left(2-s_{\mathrm{host}}\right) + \sqrt{\Delta_P}}{2\,\left(2-s_{\mathrm{cancer}}\right)} \tag{15}$$

Therefore, $\mathrm{trace}\left(\mathbf{J}^*\right)^2 - 4\det\left(\mathbf{J}^*\right) > 0$ if $\alpha > A_+$. This means that the eigenvalues are real for $\alpha \geq A_+$, and complex for $\alpha < A_+$. We now infer the stability of the equilibrium point under these two conditions (points a and b below).

a) For $\alpha \geq A_+$: Eigenvalues are real. Stability requires that the absolute value of the leading eigenvalue λ_{L} is less than one. Given that $\mathrm{trace}\left(\mathbf{J}^*\right) > 0$, the leading eigenvalue is:

$$\lambda_{\mathrm{L}} = \frac{\mathrm{trace}\left(\mathbf{J}^*\right) + \sqrt{\mathrm{trace}\left(\mathbf{J}^*\right)^2 - 4\det\left(\mathbf{J}^*\right)}}{2} > 0 \tag{16}$$

We can show that $\lambda_{\mathrm{L}} < 1$ by determining the sign of

$$\lambda_{\mathrm{L}} - 1 = \frac{\sqrt{\mathrm{trace}\left(\mathbf{J}^*\right)^2 - 4\det\left(\mathbf{J}^*\right)} - \alpha\,\left(1 + \frac{s_{\mathrm{host}}}{2-s_{\mathrm{host}}}\right)}{2} \tag{17}$$

Which has the same sign as:

$$\mathrm{trace}\left(\mathbf{J}^*\right)^2 - 4\det\left(\mathbf{J}^*\right) - \alpha^2\,\left(1 + \frac{s_{\mathrm{host}}}{2-s_{\mathrm{host}}}\right)^2 = \frac{4\,s_{\mathrm{host}}\left(-2\alpha\left(1-s_{\mathrm{cancer}}\right) - s_{\mathrm{cancer}}\right)}{(1-s_{\mathrm{cancer}})(1-s_{\mathrm{host}})} < 0 \tag{18}$$

Therefore, $\lambda_{\mathrm{L}} < 1$, meaning that the equilibrium point is stable.

b) For $\alpha < A_+$: Eigenvalues are complex, and take the form:

$$\lambda = \frac{\mathrm{trace}\left(\mathbf{J}^*\right)}{2} \pm i\frac{\sqrt{-\mathrm{trace}\left(\mathbf{J}^*\right)^2 + 4\det\left(\mathbf{J}^*\right)}}{2} \tag{19}$$

Stability requires that:

$$\sqrt{\left(\frac{\mathrm{trace}\left(\mathbf{J}^*\right)}{2}\right)^2 + \left(\frac{\sqrt{-\mathrm{trace}\left(\mathbf{J}^*\right)^2 + 4\det\left(\mathbf{J}^*\right)}}{2}\right)^2} = \sqrt{\det\left(\mathbf{J}^*\right)} < 1 \tag{20}$$

Given that $\sqrt{\det\left(\mathbf{J}^*\right)} > 0$, this means that stability requires $\det\left(\mathbf{J}^*\right) < 1$:

$$\det\left(\mathbf{J}^*\right) - 1 = \frac{-\alpha\,(2-s_{\mathrm{host}})(2-s_{\mathrm{cancer}}) - \alpha\,s_{\mathrm{host}}\,s_{\mathrm{cancer}} + s_{\mathrm{host}}\,s_{\mathrm{cancer}}}{(2-s_{\mathrm{host}})(2-s_{\mathrm{cancer}})} \tag{21}$$

Therefore, stability requires that α is higher than a threshold value A^*:

$$\alpha > A^* = \frac{s_{\mathrm{host}}\,s_{\mathrm{cancer}}}{(2-s_{\mathrm{host}})(2-s_{\mathrm{cancer}}) + s_{\mathrm{host}}\,s_{\mathrm{cancer}}} \tag{22}$$

In other words, the equilibrium point (0.5,0.5) is **unstable if** $\alpha < A^*$

Comparison between A^* and A_+: We can show that if the condition $\alpha < A^*$ is fulfilled, then $\alpha < A_+$:

$$\frac{A_+}{A^*} - 1 = \frac{U_1 + U_2}{2\,(2 - s_{\text{cancer}})\, s_{\text{host}}\, s_{\text{cancer}}} \tag{23}$$

With:

$$U_1 = s_{\text{host}}\,(1 - s_{\text{cancer}})\,(2 - s_{\text{host}})\,[(2 - s_{\text{host}})(2 - s_{\text{cancer}}) + s_{\text{host}}\, s_{\text{cancer}}] > 0 \tag{24}$$

$$\begin{aligned} U_2 = [(2 - s_{\text{host}})(2 - s_{\text{cancer}}) + s_{\text{host}}\, s_{\text{cancer}}]&\sqrt{s_{\text{host}}^2\,(1 - s_{\text{cancer}})^2\,(2 - s_{\text{host}})^2 + (2 - s_{\text{cancer}})\,(2 - s_{\text{host}})\, s_{\text{host}}\, s_{\text{cancer}}} \\ &- 2\,(2 - s_{\text{cancer}})\, s_{\text{host}}\, s_{\text{cancer}} \end{aligned} \tag{25}$$

We now determine the sign of U_2, which is the same as:

$$\begin{aligned} U_3 = [(2 - s_{\text{host}})(2 - s_{\text{cancer}}) + s_{\text{host}}\, s_{\text{cancer}}]^2 &\left[s_{\text{host}}^2\,(1 - s_{\text{cancer}})^2\,(2 - s_{\text{host}})^2 + (2 - s_{\text{cancer}})\,(2 - s_{\text{host}})\, s_{\text{host}}\, s_{\text{cancer}}\right] \\ &- 4\,(2 - s_{\text{cancer}})^2\, s_{\text{host}}^2\, s_{\text{cancer}}^2 = V_1 + V_2 \end{aligned} \tag{26}$$

With:

$$V_1 = [(2 - s_{\text{host}})(2 - s_{\text{cancer}}) + s_{\text{host}}\, s_{\text{cancer}}]^2 \left[s_{\text{host}}^2\,(1 - s_{\text{cancer}})^2\,(2 - s_{\text{host}})^2\right] > 0 \tag{27}$$

$$V_2 = [(2 - s_{\text{host}})(2 - s_{\text{cancer}}) + s_{\text{host}}\, s_{\text{cancer}}]^2\,[(2 - s_{\text{cancer}})\,(2 - s_{\text{host}})\, s_{\text{host}}\, s_{\text{cancer}}] - 4\,(2 - s_{\text{cancer}})^2\, s_{\text{host}}^2\, s_{\text{cancer}}^2 \tag{28}$$

We get:

$$V_2 = s_{\text{host}}\, s_{\text{cancer}}(2 - s_{\text{cancer}}) \left[4\,\left(2 - s_{\text{host}}\,(2 - s_{\text{host}})^2\right) s_{\text{cancer}}^2 - 8\,\left(4 - s_{\text{host}}\,(s_{\text{host}}^2 - 5\, s_{\text{host}} + 7)\right) s_{\text{cancer}} + 4\,(2 - s_{\text{cancer}})^3\right] \tag{29}$$

The polynomial function in the last term of this equation is a positive function for $0 < s_{\text{host}} < 1$ and $0 < s_{\text{cancer}} < 1$. Therefore:

$$V_2 > 0 \quad\longrightarrow\quad U_3 > 0 \quad\longrightarrow\quad U_2 > 0 \quad\longrightarrow\quad \frac{A_+}{A^*} - 1 > 0 \quad\longrightarrow\quad A^* < A_+ \tag{30}$$

The condition for unstability ($\alpha < A^*$) is true only if eigenvalues are complex. Here we showed that if the condition $\alpha < A^*$ is fulfilled, then the eigenvalues are necessarily complex ($\alpha < A_+$).
