# Supplementary material for: Transmissible cancers and the evolution of sex under the Red Queen hypothesis
Source: PLoS Biol. 2020 Nov 19;18(11):e3000916. doi: 10.1371/journal.pbio.3000916 (PMC7676742; doi:10.1371/journal.pbio.3000916)
Supplement: S2 Appendix — (PDF) [file pbio.3000916.s007.pdf]

S2 APPENDIX:
Numerical analysis of a population genetic model with similarity selection

Purpose

The Red Queen Hypothesis for the evolution of sex traditionally relies on the existence of coevolutionary cycling. Nonetheless, parasite-host interactions can still promote the evolution of sex through a separate mechanism, known as similarity selection, that does not depend on genotypic selection such as would be imposed by coevolutionary fluctuations (Agrawal, 2006). Similarity selection occurs when there is a cost to being genotypically similar to one's family members. In particular, due to the transmission of parasites among family members, this cost exists if infection compatibility is under genetic influence (e.g., a matching alleles system). Therefore, vertical transmission of parasites leading to similarity selection can be a potent force favouring the evolution of sex and recombination (Agrawal, 2006).

We here modify the three-locus population genetic model of the manuscript to investigate whether similarity selection favours the evolution of sex (as in Agrawal, 2006) in the face of neoplasia dampening coevolutionary cycling.

Including vertical transmission and similarity selection

Just like in the three-locus population genetic model presented in the manuscript, we follow the genotypic frequencies of haploid hosts and haploid cancer cells through a life cycle that consists of a census, reproduction, neoplasia (development of neocancers), and selection.

During the reproduction phase, we now keep track of the association between offspring's and mother's genotypes. In particular, assuming that there is no mutation, offspring resulting from asexual reproduction always have the same genotype as their mother. By contrast, offspring resulting from sexual reproduction may have a different genotype as their mother.

During the selection phase, we keep track of the proportions of hosts of each genotype that have been infected by transmissible cancer (via genetic matching). This corresponds to the proportion of infected mothers of each genotype at the next selection phase.

During the selection phase, we now consider that a fraction Φ of hosts encounter transmissible cancers that successfully infected their mother (if any, those transmissible cancers therefore match the mother's genotype), while the other fraction $1 - \Phi$ encounter parasites at random (as in the main analysis). Parameter Φ therefore reflects the proportion of vertical transmission that causes similarity selection.

We fix the parameter Φ, and we perform the same sensitivity analysis as in Figure 1.

Results

When we consider some extent of vertical transmission ($\Phi > 0$), a modifier allele associated with sexual reproduction can invade in an asexual population (as shown by Agrawal, 2006) (Fig. B1). We note that transmission among relatives does not dampen coevolutionary cycling as in Greenspoon and Mideo (2017); this is because we model explicitly vertical transmission by keeping track of the association between the mother-offspring genotypes.

Just like in the main analysis, even a small proportion of neocancers is sufficient to bring the coevolutionary dynamics between hosts and transmissible cancers to a halt. Nonetheless, similarity selection does not depend on coevolutionary fluctuations and favours sex (Fig. B1). Remember that we have not implemented any cost associated with sexual reproduction in this model, except the recombination load. Theoretical literature on similarity selection remains scarce, and whether similarity selection can overcome the two-fold cost of sex remains an open question. To our knowledge, three theoretical models on host-parasite interactions included similarity selection occurring via parental transmission of parasites (Agrawal, 2006; Greenspoon and M'Gonigle, 2013, 2014), and only one of those investigated its implication for sexual reproduction (Agrawal, 2006).

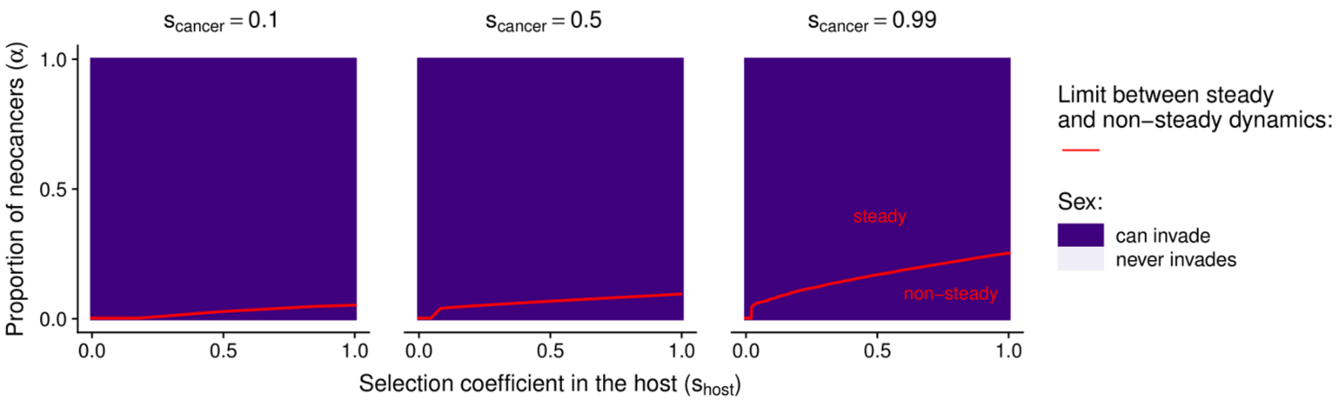

Figure B1: Coevolutionary dynamics between hosts and transmissible cancers, and evolution of sex when accounting for vertical transmission and similarity selection. Sensitivity of the population genetic models to the selection coefficients ($s_{\text{host}}, s_{\text{cancer}}$) and to the proportion of transmissible neocancers that are recently derived from the original host (α). Red lines delimit the parameter spaces leading to non-steady and steady coevolutionary dynamics. The dynamic is defined as 'steady' when the variance in genotypic frequencies over 500 time steps is below 10^{-10}. Dark purple indicates conditions under which a modifier allele associated with sexual reproduction (and with recombination, at least for one of the recombination rates tested) can invade in at least one of the 100 simulation runs. $\Phi = 0.1$.

References

Agrawal A. F., 2006. Similarity selection and the evolution of sex: Revisiting the red queen. *PLoS Biology*, 4(8):1364–1371. doi: 10.1371/journal.pbio.0040265.

Greenspoon P. B. and M'Gonigle L. K., 2013. The evolution of mutation rate in an antagonistic coevolutionary model with maternal transmission of parasites. *Proceedings of the Royal Society B: Biological Sciences*, 280(1761):20130647. doi: 10.1098/rspb.2013.0647.

Greenspoon P. B. and M'Gonigle L. K., 2014. Host-parasite interactions and the evolution of nonrandom mating. *Evolution*, 68(12):3570–3580. doi: 10.1111/evo.12538.

Greenspoon P. B. and Mideo N., 2017. Parasite transmission among relatives halts Red Queen dynamics. *Evolution*, 71(3):747–755. doi: 10.1111/evo.13157.
