# Supplementary material for: Transmissible cancers and the evolution of sex under the Red Queen hypothesis
Source: PLoS Biol. 2020 Nov 19;18(11):e3000916. doi: 10.1371/journal.pbio.3000916 (PMC7676742; doi:10.1371/journal.pbio.3000916)
Supplement: S3 Appendix — (PDF) [file pbio.3000916.s008.pdf]

S3 APPENDIX:
Analytical derivation of the epidemiological model

Purpose

We analyze the epidemiological model presented in the manuscript (Equation 4). Using basic linear algebra, we find the expression of densities at equilibrium and we show that this equilibrium state is locally stable. We also determine the expressions determining the proportion of neocancers ($\hat{\alpha}$) and the selection coefficient caused by infection by transmissible cancers ($\hat{s}_{\text{host}}$; inferred as the lifespan reduction in the host population due to infection by transmissible cancers) at equilibrium. Finally, we determine their sensitivity to changes in parameter values.

Differential equations

The following equations control the changes in densities of susceptible hosts (S), hosts that developed a neocancer by neoplasia (I_0), and hosts that are infected by a transmitted cancer (I_{T}):

$$\begin{cases} \dfrac{\mathrm{d}S}{\mathrm{d}t} = b\,(S + I_0 + I_{\text{T}})\left(1 - \dfrac{S + I_0 + I_{\text{T}}}{K}\right) - \left(\mu + \lambda_0 + \beta\,\dfrac{I_0 + I_{\text{T}}}{S + I_0 + I_{\text{T}}}\right) S \\ \dfrac{\mathrm{d}I_0}{\mathrm{d}t} = \lambda_0\,(S + \theta\, I_{\text{T}}) - \left(\mu + \nu + \theta\,\beta\,\dfrac{I_0 + I_{\text{T}}}{S + I_0 + I_{\text{T}}}\right) I_0 \\ \dfrac{\mathrm{d}I_{\text{T}}}{\mathrm{d}t} = \beta\,\dfrac{I_0 + I_{\text{T}}}{S + I_0 + I_{\text{T}}}\,(S + \theta\, I_0) - (\mu + \nu + \theta\,\lambda_0)\, I_T \end{cases} \tag{1}$$

With birth rate $b > 0$, carrying capacity $K > 0$, mortality rates $\mu > 0$ and $\nu > 0$, rate of neoplasia $\lambda_0 > 0$, transmission rate $\beta > 0$, and rate of changes in infection status $\theta \in [0, 1]$.

With the change of variable $N = S + I_0 + I_{\text{T}}$ and $I = I_0 + I_{\text{T}}$, the system of equations is equivalent to:

$$\begin{cases} \dfrac{\mathrm{d}N}{\mathrm{d}t} = b\,N\left(1 - \dfrac{N}{K}\right) - \mu\,N - \nu\,I \\ \dfrac{\mathrm{d}I}{\mathrm{d}t} = \left(\lambda_0 + \beta\,\dfrac{I}{N}\right)(N - I) - (\mu + \nu)\,I \\ \dfrac{\mathrm{d}I_0}{\mathrm{d}t} = \lambda_0\,(N - I + \theta\,(I - I_0)) - \left(\mu + \nu + \theta\,\beta\,\dfrac{I}{N}\right) I_0 \end{cases} \tag{2}$$

Below, we derive the equilibrium state of this system of equations.

Equilibrium

At equilibrium, we get:

$$\begin{cases} N^* = K\left(1 - \dfrac{\mu + P^*\nu}{b}\right) \\ I^* = P^*\,N^* \\ I_0^* = \dfrac{\lambda_0\,(1 - P^*)}{\mu + \nu} N^* \end{cases} \tag{3}$$

With P^* the prevalence of transmissible cancers at equilibrium:

$$P^* = \frac{\beta - \lambda_0 - \mu - \nu + \sqrt{(\beta - \lambda_0 - \mu - \nu)^2 + 4\beta\lambda_0}}{2\,\beta} \in [0, 1] \tag{4}$$

To get $N^* > 0$, the condition $b > \mu + P^*\nu$ must be satisfied.
If $b \leq \mu + P^*\nu$, then $N^* \leq 0$ and the host population gets extinct.

Calculation at equilibrium:

Given that $\frac{\mathrm{d}N}{\mathrm{d}t} = 0$ at equilibrium, then $b\,N^* \left(1 - \frac{N^*}{K}\right) - \mu\,N^* - \nu\,I^* = 0$. Therefore:

$$I^* = \frac{1}{\nu} \left[b \left(1 - \frac{N^*}{K} \right) - \mu \right] N^* \tag{5}$$

Given that $\frac{\mathrm{d}I}{\mathrm{d}t} = 0$ at equilibrium, then $\left(\lambda_0 + \beta\,\frac{I^*}{N^*}\right)\,(N^* - I^*) - (\mu + \nu)\,I^* = 0$, and therefore:

$$\beta\,{I^*}^2 - (\beta - \lambda_0 - \mu - \nu)\,N^* I^* - \lambda_0 {N^*}^2 = 0 \tag{6}$$

The only positive positive root of this polynomial equation is:

$$I^* = \frac{\beta - \lambda_0 - \mu - \nu + \sqrt{(\beta - \lambda_0 - \mu - \nu)^2 + 4\beta\lambda_0}}{2\,\beta}\,N^* = P^* N^* \tag{7}$$

This gives us the prevalence P^* of transmissible cancers at equilibrium:

$$P^* = \frac{I^*}{N^*} = \frac{\beta - \lambda_0 - \mu - \nu + \sqrt{(\beta - \lambda_0 - \mu - \nu)^2 + 4\beta\lambda_0}}{2\,\beta} > 0 \tag{8}$$

$P^* < 1$ because:

$$1 - P^* = \frac{1}{2\,\beta} \left[\beta + \lambda_0 + \mu + \nu - \sqrt{(\beta - \lambda_0 - \mu - \nu)^2 + 4\beta\lambda_0} \right] \tag{9}$$

and:

$$(\beta + \lambda_0 + \mu + \nu)^2 - \left((\beta - \lambda_0 - \mu - \nu)^2 + 4\beta\lambda_0 \right) = 4\,\beta\,(\mu + \nu) > 0 \tag{10}$$

By combining equations 5 and 7, we get:

$$\frac{1}{\nu} \left[b \left(1 - \frac{N^*}{K} \right) - \mu \right] N^* = P^* N^* \tag{11}$$

Which leads to the expression of N^*:

$$N^* = K \left(1 - \frac{\mu + P^* \nu}{b} \right) \tag{12}$$

And therefore to the expression of I^*:

$$I^* = P^* N^* = P^* K \left(1 - \frac{\mu + P^* \nu}{b} \right) \tag{13}$$

The host population does not get extinct if:

$$1 - \frac{\mu + P^* \nu}{b} > 0 \tag{14}$$

$$b > \mu + P^* \nu \tag{15}$$

Which is equivalent to the condition:

$$2\,\beta\,(b - \mu) - \nu\,(\beta - \lambda_0 - \mu - \nu) - \nu\,\sqrt{(\beta - \lambda_0 - \mu - \nu)^2 + 4\beta\lambda_0} > 0 \tag{16}$$

Given that $\frac{\mathrm{d}I_0}{\mathrm{d}t} = 0$ at equilibrium, then $\lambda_0\ (N^* - I^* + \theta\ (I^* - I_0^*)) - \left(\mu + \nu + \theta\,\beta\,\frac{I^*}{N^*}\right)\ I_0^* = 0$. With $I^* = P^* N^*$, we get:

$$I_0^* = \frac{\lambda_0\,(1 - P^* + \theta\,P^*)}{\mu + \nu + \theta\,(\lambda_0 + \beta P^*)}\,N^* \tag{17}$$

Which is equivalent to:

$$I_0^* = \frac{\lambda_0\,(1 - P^*)}{\mu + \nu} \times \frac{(\mu + \nu)\,(1 - P^* + \theta\,P^*)}{(1 - P^*)\,(\mu + \nu + \theta\,(\lambda_0 + \beta P^*))} \times N^* \tag{18}$$

$$I_0^* = \frac{\lambda_0\,(1-P^*)}{\mu+\nu} \times \frac{(1-P^*)\left(\mu+\nu+\theta\,\frac{(\mu+\nu)P^*}{1-P^*}\right)}{(1-P^*)\,(\mu+\nu+\theta\,(\lambda_0+\beta P^*))} \times N^* \tag{19}$$

Yet, $\frac{(\mu+\nu)P^*}{1-P^*} = \lambda_0 + \beta P^*$ because:

$$\frac{(1-P^*)\,(\lambda_0+\beta P^*)}{\mu+\nu} = \frac{\beta-\lambda_0-\mu-\nu+\sqrt{(\beta-\lambda_0-\mu-\nu)^2+4\beta\lambda_0}}{2\,\beta} = P^* \tag{20}$$

Therefore:

$$I_0^* = \frac{\lambda_0\,(1-P^*)}{\mu+\nu} N^* \tag{21}$$

Local stability

Unlike in the population genetic model analyzed in S1 Appendix, we assume that time is continuous in the epidemiological model. At the equilibrium point, the local stability can therefore be inferred from the sign of the real part of the eigenvalues of the Jacobian matrix $\mathbf{J}$ at the equilibrium point (N^*, I^*, I_0^*), the expression of which is:

$$\mathbf{J}_{\left(N^*,I^*,I_0^*\right)} = \begin{bmatrix} b-\mu-\frac{2\,b}{K}N^* & -\nu & 0 \\ \lambda_0+\beta\,P^{*2} & (\beta-\lambda_0-\mu-\nu)-2\,\beta\,P^* & 0 \\ \lambda_0+\theta\,\beta\,P^*\frac{\lambda_0(1-P^*)}{\mu+\nu} & \lambda_0\,(\theta-1)+\theta\,\beta\frac{\lambda_0(1-P^*)}{\mu+\nu} & -(\lambda_0\,\theta+\mu+\nu)-\theta\,\beta\,P^* \end{bmatrix} \tag{22}$$

Notably, the terms of the Jacobian matrix do not depend on I^* and I_0^*. They depend however on N^*, and on P^* the prevalence of transmissible cancers at equilibrium, calculated as:

$$P^* = \frac{\beta-\lambda_0-\mu-\nu+\sqrt{(\beta-\lambda_0-\mu-\nu)^2+4\beta\lambda_0}}{2\,\beta} \in [0,1] \tag{23}$$

The last column of the Jacobian matrix $\mathbf{J}_{\left(N^*,I^*,I_0^*\right)}$ is all zeros except for the element along the diagonal. This element, $-(\lambda_0\,\theta+\mu+\nu)-\theta\,\beta\,P^*$, is therefore one of the eigenvalues. This eigenvalue is negative. The remaining eigenvalues are the eigenvalues of the smaller matrix:

$$\mathbf{J}'_{\left(N^*,I^*,I_0^*\right)} = \begin{bmatrix} b-\mu-\frac{2\,b}{K}N^* & -\nu \\ \lambda_0+\beta\,P^{*2} & (\beta-\lambda_0-\mu-\nu)-2\,\beta\,P^* \end{bmatrix} \tag{24}$$

We can infer the sign of real parts of the remaining eigenvalues from the signs of the determinant and the trace of the matrix. In particular, the real parts of the remaining eigenvalues are both negative (which is the condition for equilibria to be stable) if: $\det\left(\mathbf{J}'_{(\mathbf{N^*},\mathbf{I^*},\mathbf{I_0^*})}\right) > 0$ and $\mathrm{trace}\left(\mathbf{J}'_{(\mathbf{N^*},\mathbf{I^*},\mathbf{I_0^*})}\right) < 0$.

At equilibrium, $N^* = K\left(1-\frac{\mu+P^*\nu}{b}\right)$, and we can calculate:

$$\det\left(\mathbf{J}'_{\left(N^*,I^*,I_0^*\right)}\right) = \frac{\sqrt{(\beta-\lambda_0-\mu-\nu)^2+4\beta\lambda_0}}{2\,\beta}\left[2\,\beta\,(b-\mu)-\nu\,(\beta-\lambda_0-\mu-\nu)-\nu\sqrt{(\beta-\lambda_0-\mu-\nu)^2+4\beta\lambda_0}\right] \tag{25}$$

Following the condition of existence of the equilibrium (Equation 16), the second term of this expression is positive, and therefore:

$$\det\left(\mathbf{J}'_{\left(N^*,I^*,I_0^*\right)}\right) > 0 \tag{26}$$

We also get the expression of the trace of the matrix:

$$\mathrm{trace}\left(\mathbf{J}'_{\left(N^*,I^*,I_0^*\right)}\right) = -b+\beta-\lambda_0+\nu+2\,(\nu-\beta)\,P^* \tag{27}$$

$$\begin{aligned}\mathrm{trace}\left(\mathbf{J}'_{\left(N^*,I^*,I_0^*\right)}\right) = \frac{1}{\beta}\Bigg[-\left(2\,\beta\,(b-\mu)-\nu\,(\beta-\lambda_0-\mu-\nu)-\nu\sqrt{(\beta-\lambda_0-\mu-\nu)^2+4\beta\lambda_0}\right) \\ +\,\beta\left(b-\mu-\sqrt{(\beta-\lambda_0-\mu-\nu)^2+4\beta\lambda_0}\right)\Bigg]\end{aligned} \tag{28}$$

If $b-\mu-\sqrt{(\beta-\lambda_0-\mu-\nu)^2+4\beta\lambda_0}\leq 0$, we can conclude from Equation 28 that the trace of the matrix $\mathbf{J'}_{(\mathbf{N^*},\mathbf{I^*},\mathbf{I_0^*})}$ is negative (given that the condition of existence of the equilibrium if fulfilled; Equation 16).

The trace can also be expressed as:

$$\text{trace}\left(\mathbf{J'}_{(N^*,I^*,I_0^*)}\right)=-\left(b-\mu-\sqrt{(\beta-\lambda_0-\mu-\nu)^2+4\beta\lambda_0}\right)+2\left(\nu\,P^*-\sqrt{(\beta-\lambda_0-\mu-\nu)^2+4\beta\lambda_0}\right) \tag{29}$$

As detailed below, we can show that $\nu\,P^*-\sqrt{(\beta-\lambda_0-\mu-\nu)^2+4\beta\lambda_0}<0$. Therefore, the trace is also negative if $b-\mu-\sqrt{(\beta-\lambda_0-\mu-\nu)^2+4\beta\lambda_0}>0$. Thus, the trace of the matrix $\mathbf{J'}_{(\mathbf{N^*},\mathbf{I^*},\mathbf{I_0^*})}$ is always negative under the condition of existence of the equilibrium.

Given that $\det\left(\mathbf{J'}_{(\mathbf{N^*},\mathbf{I^*},\mathbf{I_0^*})}\right)>0$ and $\text{trace}\left(\mathbf{J'}_{(\mathbf{N^*},\mathbf{I^*},\mathbf{I_0^*})}\right)<0$ under the condition of existence of the equilibrium, the real parts of the two remaining eigenvalues of the Jacobian matrix at equilibrium are both negative (just like the first eigenvalue; determined directly from the expression of the Jacobian matrix, Equation 22). **The equilibrium point is therefore locally stable**.

Proof that $\nu\,P^*-\sqrt{(\beta-\lambda_0-\mu-\nu)^2+4\beta\lambda_0}<0$ **:**

Here we use the notation $X=(\beta-\lambda_0-\mu-\nu)^2+4\beta\lambda_0$, and we aim at showing that $\nu\,P^*-\sqrt{X}<0$.
$\nu\,P^*-\sqrt{X}$ has the same sign as: $\nu^2\,{P^*}^2-X$. We can calculate:

$$\nu^2\,{P^*}^2-X=\frac{1}{4\,\beta^2}\left[(\nu-2\beta)\,(\nu+2\beta)\,\sqrt{X}^2+2\nu^2\,(\beta-\lambda_0-\mu-\nu)\,\sqrt{X}+\nu^2\,(\beta-\lambda_0-\mu-\nu)^2\right] \tag{30}$$

We call Q the polynomial function:

$$Q(x)=(\nu-2\beta)\,(\nu+2\beta)\,x^2+2\nu^2\,(\beta-\lambda_0-\mu-\nu)\,x+\nu^2\,(\beta-\lambda_0-\mu-\nu)^2 \tag{31}$$

Therefore, $\nu\,P^*-\sqrt{X}$ has the same sign as the polynomial fonction Q at $x=\sqrt{X}$.
The discriminant of this polynomial function Q is positive:

$$\Delta_Q=16\nu^2\beta^2\,(\beta-\lambda_0-\mu-\nu)^2>0 \tag{32}$$

And there are therefore two values x_1 and x_2 that are solution to the equation $Q(x)=0$:

$$x_1=\frac{-\nu\,(\beta-\lambda_0-\mu-\nu)}{\nu+2\beta} \tag{33}$$

$$x_2=\frac{-\nu\,(\beta-\lambda_0-\mu-\nu)}{\nu-2\beta} \tag{34}$$

If $\nu-2\beta\geq 0$, then we have necessarily $\beta-\lambda_0-\mu-\nu\leq 0$, and $x_1>0$, $x_2>0$, hence $x_1<x_2$. In that condition, $Q(x)<0$ if $x\in[x_1,x_2]$.
For $\nu-2\beta\geq 0$, we get:

$$x_1^2-\sqrt{X}^2=\frac{-4\beta\left[(\nu+\beta)\,(\beta-\lambda_0-\mu-\nu)^2+\lambda_0\right]}{(\nu+2\beta)^2}<0 \tag{35}$$

$$x_2^2-\sqrt{X}^2=\frac{4\beta\left[(\nu-2\beta)\left[(\nu-2\beta)\,(\mu+\nu-\beta)-(\beta-\lambda_0-\mu-\nu)\,(\beta+\lambda_0+\mu)\right]+\beta\,(\beta-\lambda_0-\mu-\nu)^2\right]}{(\nu-2\beta)^2}>0 \tag{36}$$

This means that if $\nu-2\beta\geq 0$, we get $\sqrt{X}\in[x_1,x_2]$ and $Q(\sqrt{X})<0$. Therefore, in that condition, we get $\nu\,P^*-\sqrt{X}<0$.

If $\nu-2\beta<0$, then x_1 and x_2 have opposite signs, and for any $x>0$, we get $Q(x)<0$ if $x>\max(x_1,x_2)$

For $\beta - \lambda_0 - \mu - \nu \geq 0$, we get $x_1 < 0$ and $x_2 > 0$, hence $\max(x_1, x_2) = x_2$. Yet, $\sqrt{X} > x_2$ because, in that case, we have:

$$x_2^2 - \sqrt{X}^2 = \frac{4\beta\left[(\nu-\beta)(\beta-\lambda_0-\mu-\nu)^2 - \lambda_0(\nu-2\beta)^2\right]}{(\nu-2\beta)^2} < 0 \tag{37}$$

Indeed, for $\beta - \lambda_0 - \mu - \nu > 0$, we have necessarily $\nu - \beta < 0$.
For $\beta - \lambda_0 - \mu - \nu < 0$, we get $x_1 > 0$ and $x_2 < 0$, hence $\max(x_1, x_2) = x_1$. Yet, $\sqrt{X} > x_1$ because, in that case, we have:

$$x_1^2 - \sqrt{X}^2 = \frac{-4\beta\left[(\nu+\beta)(\beta-\lambda_0-\mu-\nu)^2 + \lambda_0\right]}{(\nu+2\beta)^2} < 0 \tag{38}$$

This means that if $\nu - 2\beta < 0$, we get $\sqrt{X} > \max(x_1, x_2)$ and $Q(\sqrt{X}) < 0$. Therefore, in that condition, we get again $\nu P^* - \sqrt{X} < 0$.

Proportion of neocancers at equilibrium

At equilibrium, we calculate the proportion of neocancers as:

$$\hat{\alpha} = \frac{I_0^*}{I^*}. \tag{39}$$

Therefore:

$$\boxed{\hat{\alpha} = \frac{\lambda_0}{\mu+\nu}\left(\frac{1}{P^*} - 1\right)} \tag{40}$$

Selection coefficient due to transmissible cancers at equilibrium

At equilibrium, we infer the selection coefficient as the lifespan reduction due to the risk of being infected by transmissible cancers:

$$\boxed{\hat{s}_{\text{host}} = \frac{1}{1 + \frac{\mu}{\nu}\frac{1}{P^*}}} \tag{41}$$

Calculation:

The mean time spent in a each state (S, I_0, I_T, cf. Equation 1) is:

$$t_S = \frac{1}{\mu + \lambda_0 + \beta\frac{I^*}{N^*}} \tag{42}$$

$$t_{I_0} = \frac{1}{\mu + \nu + \theta\,\beta\frac{I^*}{N^*}} \tag{43}$$

$$t_{I_\text{T}} = \frac{1}{\mu + \nu + \theta\,\lambda_0} \tag{44}$$

As a newly infected individual (by either a neocancer or a transmitted cancer), you live:

$$\begin{cases} T_{I_0} = t_{I_0} + \dfrac{\theta\,\beta\frac{I^*}{N^*}}{\mu+\nu+\theta\,\beta\frac{I^*}{N^*}}\,t_{I_\text{T}} \\ T_{I_\text{T}} = t_{I_\text{T}} + \dfrac{\theta\,\lambda_0}{\mu+\nu+\theta\,\lambda_0}\,t_{I_0} \end{cases} \tag{45}$$

Which gives us:

$$T_{I_0} = T_{I_\text{T}} = \frac{1}{\mu+\nu} \tag{46}$$

As a new born, you live:

$$T_S = t_S + \frac{\lambda_0}{\mu + \lambda_0 + \beta \frac{I^*}{N^*}} T_{I_0} + \frac{\beta \frac{I^*}{N^*}}{\mu + \lambda_0 + \beta \frac{I^*}{N^*}} T_{I_{\mathrm{T}}} \tag{47}$$

$$T_S = \frac{1}{\mu + \lambda_0 + \beta \frac{I^*}{N^*}} \left(1 + \frac{\lambda_0 + \beta \frac{I^*}{N^*}}{\mu + \nu} \right) \tag{48}$$

Without transmissible cancers, an individuals has an average lifespan equal to $1/\mu$. The lifespan reduction due to the risk of being infected by transmissible cancers is therefore:

$$\hat{s}_{\mathrm{host}} = 1 - \frac{T_S}{1/\mu} \tag{49}$$

$$\hat{s}_{\mathrm{host}} = \frac{\nu \left(\lambda_0 + \beta \frac{I^*}{N^*} \right)}{(\mu + \nu) \left(\mu + \lambda_0 + \beta \frac{I^*}{N^*} \right)} \tag{50}$$

Yet, given that $\frac{\mathrm{d}I}{\mathrm{d}t} = 0$ at equilibrium, then $\left(\lambda_0 + \beta \frac{I^*}{N^*} \right) (N^* - I^*) - (\mu + \nu) I^* = 0$, and $\beta \frac{I^*}{N^*} = \frac{(\mu+\nu) I^*}{N^* - I^*} - \lambda_0$ and therefore:

$$\hat{s}_{\mathrm{host}} = \frac{\nu I^*}{\mu N^* + \nu I^*} \tag{51}$$

This means that the lifespan reduction due to the risk of being infected by transmissible cancers is equivalent to the relative mortality rate caused by cancer in the population.

Finally, given that $I^* = P^* N^*$ at equilibrium:

$$\hat{s}_{\mathrm{host}} = \frac{1}{1 + \frac{\mu}{\nu} \frac{1}{P^*}} \tag{52}$$

Effect of b, K and θ

While parameters b and K change the densities N^*, I^*, ${I_0}^*$ at equilibrium, they do not affect P^*, $\hat{\alpha}$ and $\hat{s}_{\mathrm{host}}$.
Parameter θ has no effect on the densities N^*, I^*, ${I_0}^*$ at equilibrium, and does not affect P^*, $\hat{\alpha}$ and $\hat{s}_{\mathrm{host}}$.

Effect of ν

On the prevalence of transmissible cancers:
We determine the effect of ν on the prevalence of transmissible cancers:

$$\frac{\partial P^*}{\partial \nu} = \frac{-P^*}{\sqrt{(\beta - \lambda_0 - \mu - \nu)^2 + 4\beta\lambda_0}} < 0 \tag{53}$$

An increase in the cancer-associated mortality rate increases the mortality rate of infected hosts, thereby decreasing the prevalence of transmissible cancers at equilibrium.

On the proportion of neocancers:
We determine the effect of ν on the proportion of neocancers at equilibrium:

$$\frac{\partial \hat{\alpha}}{\partial \nu} = \lambda_0 \left[\frac{\partial \frac{1}{\mu+\nu}}{\partial \nu} \left(\frac{1}{P^*} - 1 \right) + \frac{1}{\mu + \nu} \frac{\partial \left(\frac{1}{P^*} - 1 \right)}{\partial \nu} \right] \tag{54}$$

The cancer-associated mortality rate decreases both the densities of hosts infected by a neocancers (I_0) and hosts infected by transmitted cancer (I_{T}) at equilibrium. As shown by the signs of the terms $\frac{\partial \frac{1}{\mu+\nu}}{\partial \nu} < 0$ and $\frac{\partial \left(\frac{1}{P^*} - 1 \right)}{\partial \nu} > 0$, the cancer-associated mortality rate affects the proportion of neocancer $\hat{\alpha}$ depending on those effects on I_0 and $I_0 + I_{\mathrm{T}}$.

$$\frac{\partial \hat{\alpha}}{\partial \nu} = \frac{\lambda_0 \left[\mu + \nu - (1 - P^*) \sqrt{(\beta - \lambda_0 - \mu - \nu)^2 + 4\beta\lambda_0}\right]}{(\mu + \nu)^2 P^* \sqrt{(\beta - \lambda_0 - \mu - \nu)^2 + 4\beta\lambda_0}} \tag{55}$$

$$\frac{\partial \hat{\alpha}}{\partial \nu} = \frac{\lambda_0 \left[(\beta - \lambda_0 - \mu - \nu)^2 + 2\,\beta\,(\mu + \nu + 2\,\lambda_0) - (\beta + \lambda_0 + \mu + \nu) \sqrt{(\beta - \lambda_0 - \mu - \nu)^2 + 4\beta\lambda_0}\right]}{2\,\beta\,(\mu + \nu)^2 P^* \sqrt{(\beta - \lambda_0 - \mu - \nu)^2 + 4\beta\lambda_0}} \tag{56}$$

Yet,

$$\left[(\beta - \lambda_0 - \mu - \nu)^2 + 2\,\beta\,(\mu + \nu + 2\,\lambda_0)\right]^2 - (\beta + \lambda_0 + \mu + \nu)^2 \left((\beta - \lambda_0 - \mu - \nu)^2 + 4\beta\lambda_0\right) = 4\,\beta^2(\mu + \nu)^2 > 0 \tag{57}$$

And therefore:

$$\frac{\partial \hat{\alpha}}{\partial \nu} > 0 \tag{58}$$

A high cancer-associated mortality rate decreases the density of hosts infected by transmitted cancers relatively more than the density of hosts infected by neocancers. This makes sense because a high mortality rate of infected hosts decreases the density of hosts infected by transmitted cancers via both increased mortality and reduced transmission. Overall, increased cancer-associated mortality rate increases the proportion of neocancers.

On the selection coefficient:
The cancer-associated mortality rate has antagonistic effects on the selection coefficient due to transmissible cancers:

$$\frac{\partial \hat{s}_{\text{host}}}{\partial \nu} = \frac{\mu}{(P^*\,\nu + \mu)^2} \frac{\partial\,(\nu P^*)}{\partial \nu} \tag{59}$$

As emphasized by the term $\frac{\partial(\nu P^*)}{\partial \nu}$, a high cancer-associated mortality rate increases mortality of infected hosts, but also decreases the cancer prevalence (which increases $\hat{s}_{\text{host}}$). The balance between those antagonistic effects will determine whether increased cancer-associated mortality rate decreases or increases the selection coefficient due to transmissible cancers – i.e, whether $\frac{\partial \hat{s}_{\text{host}}}{\partial \nu} < 0$ or > 0.

$$\frac{\partial \hat{s}_{\text{host}}}{\partial \nu} = \frac{\mu\,P^*}{(P^*\,\nu + \mu)^2} \times \left(1 - \frac{\nu}{\sqrt{(\beta - \lambda_0 - \mu - \nu)^2 + 4\beta\lambda_0}}\right) \tag{60}$$

Therefore, $\frac{\partial \hat{s}_{\text{host}}}{\partial \nu}$ has the same sign as:

$$\left((\beta - \lambda_0 - \mu - \nu)^2 + 4\beta\lambda_0\right) - \nu^2 = 2\,(\mu + \lambda_0 - \beta)\,(\mu + \nu) + (\lambda_0 + \beta)^2 - \mu^2, \tag{61}$$

and $\frac{\partial \hat{s}_{\text{host}}}{\partial \nu} > 0$ for:

$$\begin{cases} \mu + \nu \leq \dfrac{\mu^2 - (\lambda_0 + \beta)^2}{2\,(\mu + \lambda_0 - \beta)}, & \text{for} \quad \mu \leq \beta - \lambda_0 \\ \mu + \nu > \dfrac{\mu^2 - (\lambda_0 + \beta)^2}{2\,(\mu + \lambda_0 - \beta)}, & \text{for} \quad \mu > \beta - \lambda_0 \end{cases} \tag{62}$$

Yet, because $\mu + \nu > \mu$, we can show that:

$$\mu > \beta - \lambda_0 \implies \mu + \nu > \frac{\mu^2 - (\lambda_0 + \beta)^2}{2\,(\mu + \lambda_0 - \beta)} \tag{63}$$

And therefore, $\frac{\partial \hat{s}_{\text{host}}}{\partial \nu} > 0$ for :

$$\begin{cases} \mu \leq \mu + \nu \leq \dfrac{\mu^2 - (\lambda_0 + \beta)^2}{2\,(\mu + \lambda_0 - \beta)}, & \text{for} \quad \mu \leq \beta - \lambda_0 \\ \mu + \nu \geq \mu, & \text{for} \quad \mu > \beta - \lambda_0 \end{cases} \tag{64}$$

$$\begin{cases} 0 \leq \nu \leq \dfrac{(\beta-\mu-\lambda_0)^2 + 4\,\lambda_0\,\beta}{2(\beta-\mu-\lambda_0)}, & \text{for } \mu \leq \beta-\lambda_0 \\ \nu \geq 0, & \text{for } \mu > \beta-\lambda_0 \end{cases} \tag{65}$$

Increased cancer-associated mortality rate increases the selection coefficient as long as the mortality rate of infected individuals ($\mu+\nu$) is low enough. Otherwise the reduction of prevalence decreases the selection coefficient due to transmissible cancers.

Effect of μ

Given that the expressions of P^* and $\hat{\alpha}$ depends on $\mu+\nu$. The parameter μ has the same effect on P^* and $\hat{\alpha}$ as parameter ν by affecting the mortality rate of infected individuals.

On the prevalence of transmissible cancers:

$$\frac{\partial P^*}{\partial \mu} = \frac{\partial P^*}{\partial \nu} < 0 \tag{66}$$

An increase in the baseline mortality rate increases the mortality rate of infected hosts, thereby decreasing the prevalence of transmissible cancers at equilibrium.

On the proportion of neocancers:

$$\frac{\partial \hat{\alpha}}{\partial \mu} = \frac{\partial \hat{\alpha}}{\partial \nu} > 0 \tag{67}$$

An increase in the baseline mortality rate increases the mortality rate of infected hosts, thereby increasing the proportion of neocancers.

On the selection coefficient:

Increased baseline mortality rate decreases the selection coefficient due to cancer by reducing both the prevalence of transmissible cancers (P^*) and the relative mortality cost associated with cancer (ν/μ).

$$\frac{\partial \hat{s}_{\text{host}}}{\partial \mu} = \frac{-\nu\,P^*}{\left(P^*\,\nu+\mu\right)^2}\left[1+\frac{\mu}{\sqrt{\left(\beta-\lambda_0-\mu-\nu\right)^2+4\beta\lambda_0}}\right] < 0 \tag{68}$$

Effect of λ_0

On the prevalence of transmissible cancers:

We determine the effect of λ_0 on the prevalence of transmissible cancers:

$$\frac{\partial P^*}{\partial \lambda_0} = \frac{1}{2\,\beta\,\sqrt{\left(\beta-\lambda_0-\mu-\nu\right)^2+4\beta\lambda_0}}\left[\beta+\lambda_0+\mu+\nu-\sqrt{\left(\beta-\lambda_0-\mu-\nu\right)^2+4\beta\lambda_0}\right] \tag{69}$$

Yet,

$$\left(\beta+\lambda_0+\mu+\nu\right)^2 - \left(\left(\beta-\lambda_0-\mu-\nu\right)^2+4\beta\lambda_0\right) = 4\,\beta\,(\mu+\nu) > 0 \tag{70}$$

Therefore:

$$\frac{\partial P^*}{\partial \lambda_0} > 0 \tag{71}$$

The rate of neoplasia increases the prevalence of transmissible cancers at equilibrium.

On the proportion of neocancers:
We determine the effect of λ_0 on the proportion of neocancers at equilibrium:

$$\frac{\partial \hat{\alpha}}{\partial \lambda_0} = \frac{1}{\mu + \nu} \left[\left(\frac{1}{P^*} - 1 \right) + \lambda_0 \frac{\partial \left(\frac{1}{P^*} - 1 \right)}{\partial \lambda_0} \right] \tag{72}$$

The rate of neoplasia increases both the densities of hosts infected by a neocancers (I_0) and hosts infected by transmitted cancer (I_{T}) at equilibrium. As shown by the sign of the terms $\frac{1}{P^*} - 1 > 0$ and $\lambda_0 \frac{\partial \left(\frac{1}{P^*} - 1 \right)}{\partial \lambda_0} < 0$, the rate of neoplasia affects the proportion of neocancer $\hat{\alpha}$ depending on those effects on I_0 and $I_0 + I_{\mathrm{T}}$.

$$\frac{\partial \hat{\alpha}}{\partial \lambda_0} = \frac{1 - P^*}{2\,\beta\,(\mu + \nu)\,{P^*}^2 \sqrt{(\beta - \lambda_0 - \mu - \nu)^2 + 4\beta\lambda_0}} \left[(\beta - \lambda_0 - \mu - \nu)^2 + 2\,\beta\,\lambda_0 + (\beta - \lambda_0 - \mu - \nu) \sqrt{(\beta - \lambda_0 - \mu - \nu)^2 + 4\beta\lambda_0} \right] \tag{73}$$

Yet,

$$\left[(\beta - \lambda_0 - \mu - \nu)^2 + 2\,\beta\,\lambda_0 \right]^2 - (\beta - \lambda_0 - \mu - \nu)^2 \left((\beta - \lambda_0 - \mu - \nu)^2 + 4\beta\lambda_0 \right) = 4\,\beta^2\,{\lambda_0}^2 > 0 \tag{74}$$

And therefore:

$$\frac{\partial \hat{\alpha}}{\partial \lambda_0} > 0 \tag{75}$$

A high rate of neoplasia increases the density of hosts infected by neocancers relatively more than the density of hosts infected by transmitted cancers. Overall, increased rate of neoplasia increases the proportion of neocancers.

On the selection coefficient:
A high rate of neoplasia increases the selection coefficient due to transmissible cancers by increasing the prevalence:

$$\frac{\partial \hat{s}_{\text{host}}}{\partial \lambda_0} = \frac{\mu}{\nu \left(1 + \frac{\mu}{\nu} \frac{1}{P^*} \right)^2 {P^*}^2} \frac{\partial P^*}{\partial \lambda_0} > 0 \tag{76}$$

Effect of β

On the prevalence of transmissible cancers:
We determine the effect of β on the prevalence of transmissible cancers:

$$\frac{\partial P^*}{\partial \beta} = \frac{1}{2\,\beta^2 \sqrt{(\beta - \lambda_0 - \mu - \nu)^2 + 4\beta\lambda_0}} \left[(\lambda_0 + \mu + \nu) \sqrt{(\beta - \lambda_0 - \mu - \nu)^2 + 4\beta\lambda_0} - \left((\lambda_0 + \mu + \nu)^2 + \beta\,(\lambda_0 - \mu - \nu) \right) \right] \tag{77}$$

Yet,

$$(\lambda_0 + \mu + \nu)^2 \left((\beta - \lambda_0 - \mu - \nu)^2 + 4\beta\lambda_0 \right) - \left((\lambda_0 + \mu + \nu)^2 + \beta\,(\lambda_0 - \mu - \nu) \right)^2 = 4\,\beta^2 \lambda_0\,(\mu + \nu) > 0 \tag{78}$$

Therefore:

$$\frac{\partial P^*}{\partial \beta} > 0 \tag{79}$$

The transmission rate increases the prevalence of transmissible cancers at equilibrium.

On the proportion of neocancers:
We determine the effect of β on the proportion of neocancers at equilibrium:

$$\frac{\partial \hat{\alpha}}{\partial \beta} = -\frac{\lambda_0}{(\mu + \nu){P^*}^2} \frac{\partial P^*}{\partial \beta} < 0 \tag{80}$$

A high transmission rate increases the density of hosts infected by transmitted cancers. Therefore, increased transmission rate decreases the proportion of neocancers.

On the selection coefficient:
A high transmission rate increases the selection coefficient due to transmissible cancers by increasing the prevalence:

$$\frac{\partial \hat{s}_{\text{host}}}{\partial \beta} = \frac{\mu}{\nu\left(1 + \frac{\mu}{\nu}\frac{1}{P^*}\right)^2 {P^*}^2} \frac{\partial P^*}{\partial \beta} > 0 \tag{81}$$
