# Supplementary material for: Transmissible cancers and the evolution of sex under the Red Queen hypothesis
Source: PLoS Biol. 2020 Nov 19;18(11):e3000916. doi: 10.1371/journal.pbio.3000916 (PMC7676742; doi:10.1371/journal.pbio.3000916)
Supplement: S4 Appendix — (PDF) [file pbio.3000916.s009.pdf]

S4 APPENDIX:
Numerical analysis of an epidemiological model with two types of cancers

Purpose

We modify the epidemiological model from the manuscript (Equation 4) to account for distinct types of transmissible cancers differing genetically (such as in the population genetic model we analyzed). Here we consider that there are two types of transmissible cancers that originate from different types of hosts (e.g., with different genetic makeup). We assume that hosts are susceptible only to the cancerous line that is originally derived from an host of their own type (because this cancerous line has the same genetic make up than their own and is not recognized as non-self).

We show below that this model has the same behaviour as the epidemiological model analyzed in the main text. In particular, we show that there is no coevolutionary cycling in this epidemiological model where time is assumed to be continuous (unlike in our population genetic model). We also highlight that by ignoring diversity in cancerous strains, we underestimated the proportion of neocancers and we overestimated the strength of selection caused by transmissible cancers. The epidemiological constraints caused by the coexistence of multiple cancerous lines therefore reduce even further the conditions under which transmissible cancers can promote the evolution of sex.

Differential equations

The following equations control the changes in densities of those two types of hosts. Again, we follow the densities of susceptible hosts (S_1 and S_2), hosts that developed a neocancer by neoplasia (I_{01} and I_{02}), and hosts that are infected by a transmitted cancer ($I_{\mathrm{T}1}$ and $I_{\mathrm{T}2}$):

$$\begin{cases} \dfrac{\mathrm{d}S_1}{\mathrm{d}t} = b\,(S_1 + I_{01} + I_{\mathrm{T}1}) \left(1 - \dfrac{N}{K}\right) - \left(\mu + \lambda_0 + \beta\,\dfrac{I_{01} + I_{\mathrm{T}1}}{N}\right) S_1 \\ \dfrac{\mathrm{d}I_{01}}{\mathrm{d}t} = \lambda_0\ (S_1 + \theta\, I_{\mathrm{T}1}) - \left(\mu + \nu + \theta\,\beta\,\dfrac{I_{01} + I_{\mathrm{T}1}}{N}\right) I_{01} \\ \dfrac{\mathrm{d}I_{\mathrm{T}1}}{\mathrm{d}t} = \beta\,\dfrac{I_{01} + I_{\mathrm{T}1}}{N}\ (S_1 + \theta\, I_{01}) - (\mu + \nu + \theta\,\lambda_0)\ I_T \\ \dfrac{\mathrm{d}S_2}{\mathrm{d}t} = b\,(S_2 + I_{02} + I_{\mathrm{T}2}) \left(1 - \dfrac{N}{K}\right) - \left(\mu + \lambda_0 + \beta\,\dfrac{I_{02} + I_{\mathrm{T}2}}{N}\right) S_2 \\ \dfrac{\mathrm{d}I_{02}}{\mathrm{d}t} = \lambda_0\ (S_2 + \theta\, I_{\mathrm{T}2}) - \left(\mu + \nu + \theta\,\beta\,\dfrac{I_{02} + I_{\mathrm{T}2}}{N}\right) I_{02} \\ \dfrac{\mathrm{d}I_{\mathrm{T}2}}{\mathrm{d}t} = \beta\,\dfrac{I_{02} + I_{\mathrm{T}2}}{N}\ (S_2 + \theta\, I_{02}) - (\mu + \nu + \theta\,\lambda_0)\ I_T \end{cases} \tag{1}$$

With $N = S_1 + I_{01} + I_{\mathrm{T}1} + S_2 + I_{02} + I_{\mathrm{T}2}$.

With birth rate $b > 0$, carrying capacity $K > 0$, mortality rates $\mu > 0$ and $\nu > 0$, rate of neoplasia $\lambda_0 > 0$, transmission rate $\beta > 0$, and rate of changes in infection status $\theta \in [0, 1]$.

Numerical analysis

We could not analyze the above model with analytical derivations. Instead, we analyzed it numerically.

We implemented initial differences in prevalence between the two cancer strains by drawing initial densities (S_1, S_2, I_{01}, I_{02}, $I_{\mathrm{T}1}$, $I_{\mathrm{T}2}$)) from a uniform distribution across the range [1, $K/6$]. Using Julia (version 1.0.1), we then ran simulations until time $t = 5,000$.

We tested the same parameter values as in Figure 4 in the manuscript (with $b = 1.0$, $K = 500$, $\theta = 0$). For each combinations of parameters, we ran two replicates characterized by different initial densities to investigate the existence

of an unstable equilibrium state.

At the end of the simulation, we calculated the prevalence as:

$$\text{Prevalence} = \frac{I_{01} + I_{02} + I_{\text{T}1} + I_{\text{T}2}}{N} \tag{2}$$

We also calculated the proportion of neocancers ($\hat{\alpha}$) as:

$$\hat{\alpha} = \frac{I_{01} + I_{02}}{I_{01} + I_{02} + I_{\text{T}1} + I_{\text{T}2}} \tag{3}$$

Finally, we assessed the selection coefficient caused by transmissible cancers ($\hat{s}_{\text{host}}$) as the relative mortality rate caused by cancer in the population (cf. Equation 51 in S3 Appendix):

$$\hat{s}_{\text{host}} = \frac{(I_{01} + I_{02} + I_{\text{T}1} + I_{\text{T}2})\,\nu}{(I_{01} + I_{02} + I_{\text{T}1} + I_{\text{T}2})\,(\mu + \nu) + (S_1 + S_2)\,\mu} \tag{4}$$

Results

Nature of the equilibrium state:

If the system was characterized by an unstable equilibrium, we would observe variations among the replicate runs that differed in their initial state (either because of cycling or chaotic behaviours, or because of convergence towards distinct stable equilibria). Here, however, we get the same $\hat{\alpha}$ and $\hat{s}_{\text{host}}$ values at the end of the two replicate ran for each combination of parameters (Fig. D1). Therefore, we can conclude that the system is characterized by only one stable equilibrium, just like the simpler system we analyzed in the main text. Overlapping generations and epidemiological dynamics (with changes in parasite prevalence) are probably the causes behind this dampening of coevolutionary cycling among hosts and cancer strains (as shown in other models; e.g., May and Anderson, 1983; Beck, 1984; Kouyos et al., 2007; MacPherson and Otto, 2018, cited in our manuscript).

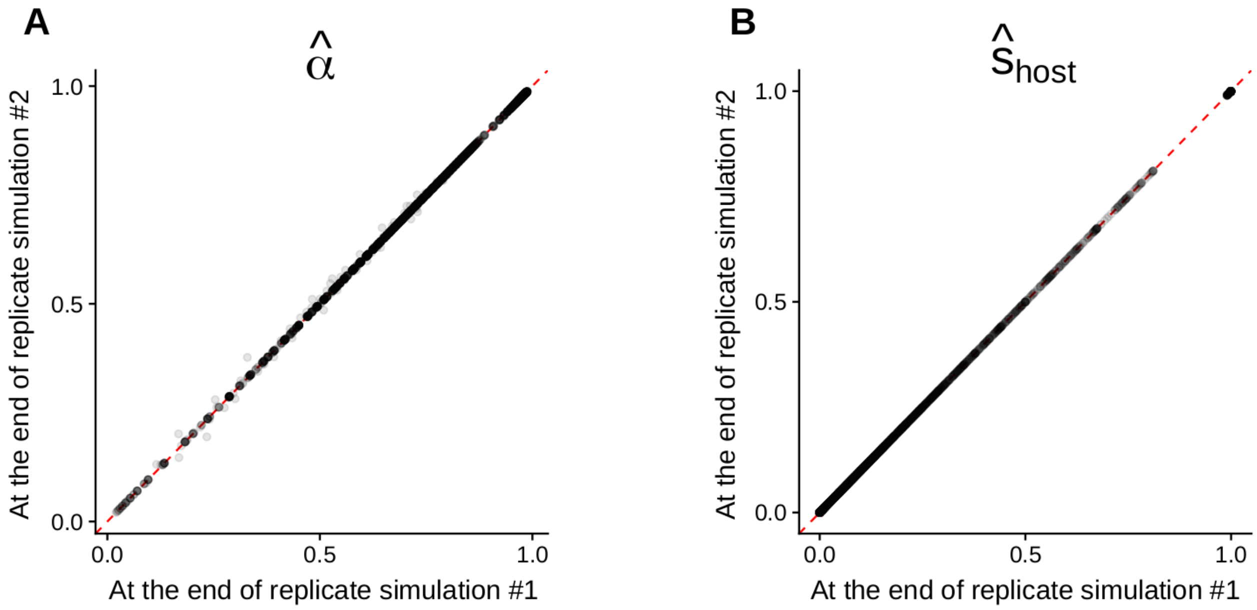

Figure D1: Comparison of the output ($\hat{\alpha}$ and $\hat{s}_{\text{host}}$ values at the end of the simulations) of two replicates differing by their initial conditions. We implement point transparency to ignore errors due to the numerical approximation inherent to the differential equations solver. Parameter values as in Fig. D2.

Effects of epidemiological parameters:

Epidemiological parameters have similar effects on the proportion of neocancers ($\hat{\alpha}$) and the selection coefficient caused by transmissible cancers ($\hat{s}_{\text{host}}$) at equilibrium than in the original model with only one type of transmissible cancer (Fig. D2

vs. Fig. 4A-B). Nonetheless, the prevalence of cancer is particularly low when we account for two types of transmissible cancers (Fig. D3A) because the transmission rate is lower (given that hosts can avoid infection by half of the cancerous lines). This is particularly the case when the rate of neoplasia (λ_0) is low (Fig. D3A). This leads to a high proportion of neocancers (Fig. D3B) and a low selection coefficient caused by transmissible cancers (Fig. D3C) compared to the situation with only one type of transmissible cancers. This indirect effect on $\hat{\alpha}$ and $\hat{s}_{\text{host}}$ driven by changes in prevalence was highlighted in the original model (cf. Fig. 3).

Therefore, the epidemiological constraints caused by the coexistence of multiple cancerous lines constrain even further the conditions under which transmissible cancers can promote the evolution of sex.

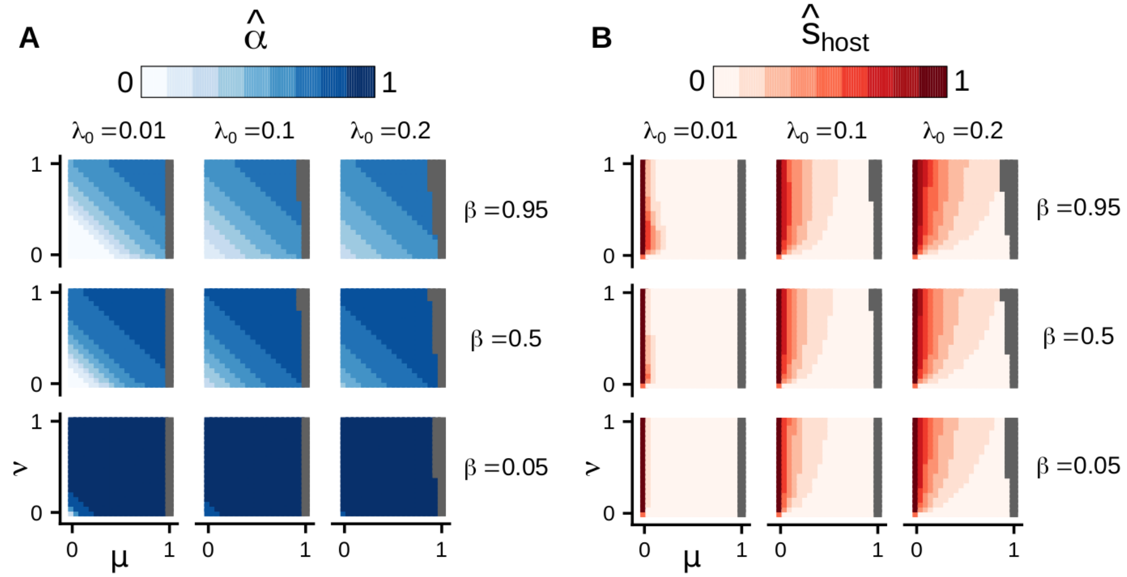

Figure D2: Effects of epidemiological parameters on $(\hat{\alpha}, \hat{s}_{\text{host}})$ at the end of the simulation when we account for two types of transmissible cancers. We represent in gray the conditions under which $N < 1$ at the end of the simulation – i.e., we can thus assume that the host population gets extinct for these combinations of parameters. Parameter values: $b = 1.0$, $K = 500$, $\theta = 0$. Note that we get the exact same results when we implement other values of parameters b, K or θ (not shown).

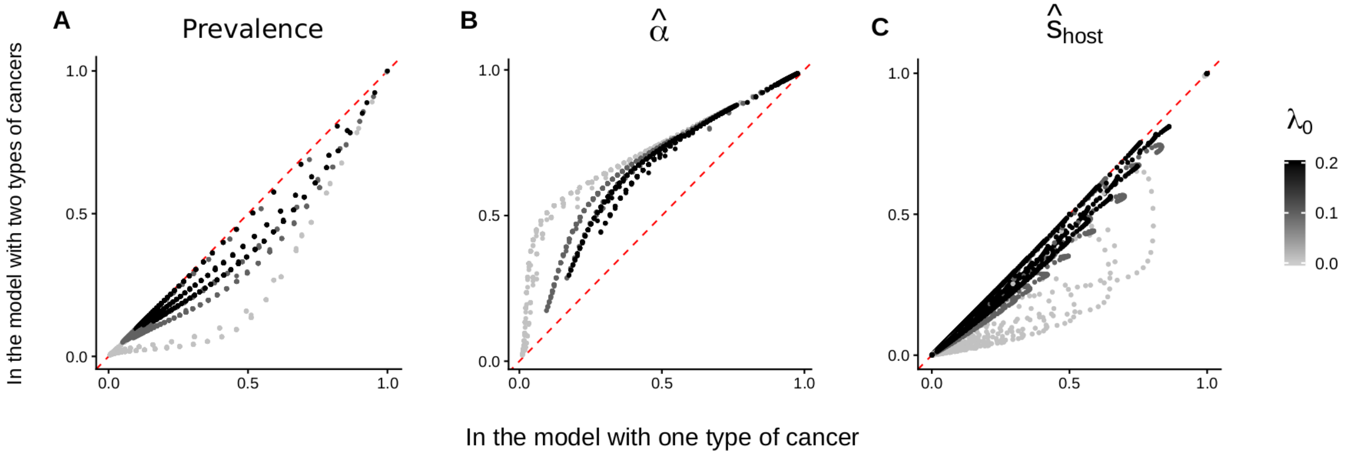

Figure D3: Comparison with the situation with only one type of cancer. We compare the output of this model with the values from the model where we considered only type of cancer. The gray scale represents the value of λ_0 (rate of neoplasia) implemented. Parameter values as in Fig. D2.

References

Beck K., 1984. Coevolution: mathematical analysis of host-parasite interactions. *Journal of Mathematical Biology*, 19(1):63–77. doi: 10.1007/BF00275931.

Kouyos R. D., Salathé M., and Bonhoeffer S., 2007. The Red Queen and the persistence of linkage-disequilibrium oscillations in finite and infinite populations. *BMC Evolutionary Biology*, 7:211. doi: 10.1186/1471-2148-7-211.

MacPherson A. and Otto S. P., 2018. Joint coevolutionary-epidemiological models dampen Red Queen cycles and alter conditions for epidemics. *Theoretical Population Biology*, 122:137–148. doi: 10.1016/j.tpb.2017.12.003.

May R. M. and Anderson R. M., 1983. Epidemiology and genetics in the coevolution of parasites and hosts. *Proceedings of the Royal Society B: Biological Sciences*, 219(1216):281–313. doi: 10.1098/rspb.1983.0075.
