# Supplementary material for: Transmissible cancers and the evolution of sex under the Red Queen hypothesis
Source: PLoS Biol. 2020 Nov 19;18(11):e3000916. doi: 10.1371/journal.pbio.3000916 (PMC7676742; doi:10.1371/journal.pbio.3000916)
Supplement: Attachment — Submitted filename: ResponseToReviewers.pdf [file pbio.3000916.s011.pdf]

Response Letter

Response to the Editors

In light of the reviews (below), we will welcome re-submission of a revised version that takes into account the reviewers' comments. Our Academic Editor agrees with reviewer 1 that you should ideally consider an open-ended model where many loci co-evolve; however, as this could involve extensive additional work, we have decided to make it optional but not essential. Our Academic Editor suggests that the revision should consider vertical transmission (which should not be hard) and also at least discuss how facultative sex might evolve towards obligate sex. We cannot make any decision about publication until we have seen the revised manuscript and your response to the reviewers' comments. Your revised manuscript is also likely to be sent for further evaluation by the reviewers.

- We thank the Editors and the Academic Editor for handling our manuscript. We are pleased that all Referees approved our general aim of investigating formally the implications of transmissible cancers for the evolution of sexual reproduction. We have carefully revised our manuscript following their constructive criticisms. Our main revisions are as follows:
- We now also consider a case with more loci; this shows that increasing the number of loci dampens Red Queen dynamics and therefore inhibits the evolution of sex (in line with Otto & Nuismer 2004, *Science* 304(5673):1018-1020), in particular when neoplasia occurs. We also now discuss the implication of “arm race” dynamics for the evolution of sexual reproduction.
- We now model the evolution of obligate sex from facultative sex, and our results remained unchanged.
- A new supplementary analysis confirms that vertical transmission of transmissible cancers can promote the evolution of sex through a mechanism called similarity selection (Agrawal 2006, *PloS Biology* 4(8):e265), which differs fundamentally from Red Queen in that it does not depend on coevolutionary fluctuations. Given that neoplasia dampens fluctuating selection, transmissible cancers are therefore more likely to favour the evolution of sex via similarity selection rather than via fluctuating Red Queen dynamics. We modified our title, our abstract and our discussion in the light of this new result.
- In what follows, page and line numbers refer to the revised document without track changes.

Response to Reviewer #1 (signed: Joanna Masel)

This manuscript is an extended rebuttal of the novel hypothesis recently proposed in an essay by Thomas et al. in PLoS Biology, that protection from transmissible cancer drove the evolution of obligate sexuality as animals evolved to become multicellular. I agree with the authors of the current manuscript that the proposal of Thomas et al. should have been accompanied by a mathematical demonstration of proof of principle. However, the current manuscript attempts to provide a disproof of principle, which is always a trickier affair, as changing any number of assumptions might change the outcome, and proof of principle requires only one plausible scenario to be found.

- We thank Prof. Joanna Masel for her constructive criticisms. We agree that it is harder to disprove ("X can never happen") than prove ("X can, at least sometimes, happen"). Given that sex does *sometimes* evolve in our models (but under restricted circumstances), one could say that our goal cannot be stated to be a complete "disproof" of the possibility (or if that was the goal, then we would have failed). But it is correct to say, like Prof. Joanna Masel points out, that this state of affairs makes it important to evaluate if we have looked at best-case scenarios or worst-case scenarios for the mechanisms to operate. We clearly won't claim this is the last word; rather it is the first formal investigation of the implication of transmissible cancers for the evolution of sex. We first did that only for the Red Queen hypothesis, now we have expanded this to similarity selection (see below).
- We are therefore glad to conduct many additional analyses suggested by the Referees. As these became rather numerous, we did not do absolutely everything that was suggested. We did not switch to modeling "arm race" dynamics as suggested by Prof. Joanna Masel (see explanation below), but we (i) increased the number of interaction loci as well as (ii) considered the transition from facultative to obligate sex. Also, following one of Dr. Frédéric Thomas' suggestions (that echoes with Prof. Joanna Masel's remark: *"sexual progeny are almost always different from their parents"*), we (iii) investigated the implications of the vertical transmission of cancerous cells for the evolution of sex (i.e., when transmissible cancers are transmitted vertically from parents to offspring). Of these, (iii) proves to be the most important change to our original assumptions. If cancer can be vertically transmitted, one moves away from the Red Queen framework (this is the reason why we treat this situation in a supplementary analysis), and this made our model recover the perhaps underappreciated findings of similarity selection favouring sex (Agrawal 2006, *PloS Biology* 4(8):e265). Evoking similarity selection makes cancer a more potent force favouring sexual reproduction, compared with selection driven by fluctuating Red Queen dynamics. The importance of vertical transmission was pointed out by Thomas et al. 2019, but the connection to similarity selection was not; our new model's ability to link the current discussion to earlier work should hopefully make our manuscript even more stimulating for other complementary research on this topic.

My first major concern is with a modeling assumption that is widespread within the host-parasite coevolution theory community, but utterly baffling to someone like myself outside that who comes in grounded in molecular evolution data. This assumption is that only a small number of loci (two in this manuscript) mediate the host-parasite interaction, that fluctuating selection dynamics occur within this extremely restricted genotype space, and that it is lags within these fluctuations that drive the advantage of host sex in evolving away from parasites. The alternative "arms race" dynamic (see eg doi:10.1111/j.1420-9101.2008.01598.x for discussion) envisages open-ended evolution in many more loci, without the return to previous genotypes that characterizes fluctuations in more restricted genotype spaces that have nowhere else to go. As an outsider, I do not understand why fluctuating selection dynamics have received so much more theoretical attention than arms races; molecular studies of host-parasite interactions show adaptation occurring across vast tracts of the host genome (see eg the work of David Enard). In the context of this manuscript, it seems to me that a much larger genotype space, such that sexual progeny are

almost always different from their parents, would be closer to Fig. 1B of Thomas et al., and must be considered before their hypothesis is dismissed.

- In the literature on host-parasite interaction, the implication of fluctuating selection dynamics for the evolution of sex has indeed received much more attention than arm race dynamics (where hosts and parasites continually accumulate adaptive mutations). This may to some extent reflect vagaries of history, as early models required fluctuating selection for sex to be maintained, and the ability of host-parasite interactions to produce such dynamics captured researchers' attention (Hamilton 1980, *Oikos* 35(2):282-290). Our study looks primarily at Red Queen dynamics, as this was the suggestion of the original study that prompted ours: quoting Thomas et al., "From a theoretical perspective, our hypothesis could be tested through developing new theoretical frameworks based on mathematical models so far used to elucidate the "Red Queen" hypothesis […]". Adopting Red Queen as the basic framework requires the least deviation from the tradition of host-parasite evolution of sex models, which facilitates comparison to earlier models; if we had switched from Red Queen to arms races, it would be unclear if it was the switch to a cancer context, or the switch in the dynamic assumptions, that is responsible for any differences between earlier results and ours. That said, we believe that future models on arms race dynamics could be exciting, and in the Discussion, we refer to this: we explicitly state that sex could speed up the development of immunity to lineages of transmissible cancers (page 17, lines 419-423).
- Encouraged by the reviewer's sentiment, we also took the opportunity to test the robustness of our results to a change in the number of loci mediating the interaction between hosts and transmissible cancers (Figure S3; page 8, lines 196-199). We confirm an earlier finding showing that under the Red Queen hypothesis, sex is favoured mostly within a restricted genotypic space (Otto & Nuismer 2004, *Science* 304(5673):1018-1020). We also show that neoplasia dampens coevolutionary cycling even when considering more than two interaction loci.
- Following one of Dr. Frédéric Thomas' suggestions, we considered vertical transmission in a supplementary analysis. This allowed us to confirm that similarity selection can favour sex (with sexual progeny being different from their parents) even without fluctuating selection (Agrawal 2006, *PloS Biology* 4(8):e265) (Appendix B; page 8, lines 215-222). This gives credit to one hypothesis formulated by Thomas et al.'s opinion piece that we did not model in the initial version of our manuscript, and that involves a different process as in standard Red Queen models. We modified our title and abstract accordingly (page 2, lines 13-16).

My second major concern is that my reading of Thomas et al. is that their hypothesis applies to the transition from facultatively sexual reproduction to obligately sexual reproduction. While Thomas et al. is in places sloppily written on this point (another reason that a formal model should have been required in that work to make it clearer), I nevertheless think this is still the only reasonable reading. But the current manuscript models the transition from zero sex to obligate sex, even while it makes critical remarks about sex being an ancestral trait. It would be more appropriate to model the transition from rare sex to obligate sex.

- We agree that it is not always clear which transition Thomas et al. have in mind (they do mention facultative vs. obligate sex, but then go back to talking about the "challenges faced by asexual metazoans". Regardless of how clear the original writing was (or wasn't), it appears worthwhile to expand the analysis from the "obligate asexual -> obligate sexual" transition to one "facultative sexual -> obligate sexual", since sexual reproduction indeed is an ancestral trait for metazoans. The revised version of the manuscript includes the latter approach in a supplementary analysis. The results remain qualitatively and quantitatively similar to our original analysis (Figure S2; page 8, lines 195-196).

Because of these two major concerns, I do not find that the principle proposed by Thomas et al. has been satisfactorily disproved in this work. Major revisions to address these two concerns has

the potential to change the conclusions of this work. Minor revisions to acknowledge without addressing the concerns would substantially reduce the impact of the work.

- We hope that we addressed Prof. Joanna Masel's concerns. Our revised study emphasizes that transmissible cancers are unlikely to favour the evolution of obligate sex via fluctuating selection dynamics (as predicted under the classical Red Queen hypothesis for the evolution of sex), but, importantly, other mechanisms may come into play. We now particularly highlight the role of similarity selection, for which we produce a new model, which we believe to be an exciting addition that improves the value of this manuscript; indeed, as predicted by Prof. Joanna Masel, the 'potential to change the conclusions' indeed happened, and this also strengthens the link between our work and past literature. We believe this and the other new analyses (e.g. number of loci) elevate the changes so that they can be considered 'major' rather than 'minor' — even if some potential additional effects (particularly arm race dynamics) are discussed rather than explicitly modelled.

Response to Reviewer #2 (signed: Frederic Thomas)

Few months ago, I proposed (Thomas et al. 2019) with few colleagues in a verbal paper a novel hypothesis to explain the evolution of sexual reproduction. Our hypothesis suggested that transmissible cancer cells could have played, and would still play, a major role for the evolution and maintenance of sexual reproduction in the living world. In this paper, Aubier et al. explored the conditions that are required for this hypothesis to be plausible. Their main conclusion is that the conditions for transmissible cancers to select for sex are most favorable if transmissible cancers exist in a situation with a low rate of neoplasia, a high transmission rate and an intermediate cancer-associated mortality rate in hosts with a low baseline mortality rate. Because these conditions are very particular, they conclude that transmissible cancers do not easily select for sexual reproduction.
I found this paper excellent, timely and well written. I am also glad to see that our hypothesis had stimulated other scientists, and yielded beautiful work like this one.
I suggested Minor Revisions, but in fact it is between minor and major, I think.

- We thank Dr. Frederic Thomas for his very positive feedback. In the revised version of our manuscript, we emphasize the limitations of our study on the implications of fluctuating coevolutionary dynamics with transmissible cancers for the evolution of sex (1) by providing and additional analysis showing that similarity selection caused by vertical transmission remains a potent evolutionary force favouring sex, and (2) by discussing further other possible mechanisms that may favour the evolution of sex in the case of infection by transmissible cancers. We hope that we addressed his concerns successfully.

As authors acknowledge themselves at the end of their ms, they only considered horizontal transmission while we also suggested in Thomas et al. 2019 that vertical transmission could exert a major role, even a higher role than horizontal one. Thus, the correction I would ask, both to be fair and also to stimulate other complementary researches, is in their title. In my view, it should be something like : "Horizontal transmission of malignant cells does not easily select for sexual reproduction" (instead of Transmissible cancers do not easily select for sexual reproduction). I would REALLY appreciate if the authors could change their title accordingly.
In addition to the title problem, it is also relevant from a biological point of view. Because the model only assumes horizontal transmission, it clearly removed a key difference between sexual and asexual reproducers in respect of transmissible cancers. I would expect rare vertical transmission when individuals reproduce sexually and almost invariable vertical transmission during asexual reproduction, which could potentially turn this whole model upside down. I also believe that implementing vertical transmission in the model is key and would not be difficult (maybe I am wrong?): an offspring would acquire the transmissible cancer of the parent(s) if it has the same genotype at the interaction loci as the parent with transmissible cancer (only in case some sort of parental care (e.g. gestation/post-embryonic care) is presumable, due to physical proximity (maybe not even applicable if we talk about early multicellular organisms). On the other hand, asexual reproduction would result in vertical transmission, maybe with a given rate, in hydra for instance almost all buds of cancerous hydra develop cancer.

- In the initial submission, we only considered horizontal transmission of cancerous cells for the sake of consistency with the theoretical literature on the Red Queen hypothesis. Fluctuating coevolutionary dynamics underpin the Red Queen hypothesis for the evolution of sex, and our results show that the formation of transmissible cancers can dampen (perhaps counterintuitively) those coevolutionary fluctuations. Our original emphasis on the Red Queen directly follows the original paper's (Thomas et al. 2019) encouragement for researchers to produce models for this process in the context of cancer, a decision that we made explicit both in the title and in the abstract (page 2, lines 13-16).
- Nonetheless, Dr. Frédéric Thomas' remark made us think more about the implication of vertical transmission for the evolution of sex. This led us to put the findings in context of a paper on the topic of similarity selection (Agrawal 2006, *PloS Biology* 4(8):e265). Agrawal's paper emphasizes that vertical transmission has the potential to generate a selective force

that has also the potential of favouring sex and recombination, while being distinct from directional selection (created by coevolutionary fluctuations in standard Red Queen settings). We conducted an additional analysis, where we modified our population genetic model to account for vertical transmission of cancerous cells (from mother to offspring). This confirms that similarity selection remains a potent force favouring sex even if neoplasia dampens coevolutionary fluctuations (Appendix B; page 8, lines 215-222). Altogether, our main result and this additional analysis should stimulate other complementary research focusing on vertical transmission of cancers, while we maintain that horizontal transmission of cancers does not easily select for sex. We modified our title, our abstract (page 2, lines 13-16) and our discussion (pages 17-18, lines 426-441) accordingly.

Also the model assumes no evolution of anti-cancer mechanisms, which is another key difference between sexual and asexual reproducers. It is clearly articulated in numerous articles, that sexual reproduction accelerates adaptive evolution (see e.g. Goddard 2016 Nature), so once a transmissible cancer is present in the population sexual reproduction should accelerate the development of immunity/suppression. Even in early stages of such adaptations, sexually reproducing individuals might live a little bit longer than asexuals after acquiring cancer, which might result in their slightly higher fitness. There are indications that even Devil Facial Tumor Disease can accelerate selection in immune- and tumor-related genes, which could arguably be much slower in asexually reproducing species, e.g. in the case of Devils and DFTD, selection of defence mechanisms against the cancer have rapidly evolved in response to the epidemic (4-6 generations). Surely this selection would be much slower or perhaps not existing in the case asexual reproduction. This component might be harder to implement in the model, but should be clearly indicated in the ms as a shortfall of the work I believe. Perhaps authors should acknowledge this and suggest that the results should be interpreted with caution. If the model assumes no evolution of anticancer mechanisms then is difficult to put the results in the context of our paper.

- We agree that we have not been able to include every possible mechanism in these models. This remark aligns with Prof. Joanna Masel's first major comment about the implication of "arm race" dynamics for the evolution of sex, in which case sex and recombination is likely to accelerate adaptation.
- We now explicitly state that in our study we have not included other evolutionary processes that may favour the evolution of sexual reproduction in the case of infection by transmissible cancers, and discuss arms races in particular (page 17, lines 419-425).

I was a bit confused with the epidemiological model and how they modelled beta. In the supp material they say that beta increases the prevalence of transmissible cancers at equilibrium but is this fully independent of mortality and birth rates?

- The parameter beta controls the rate of transmission (and is classically referred to as 'transmission rate' in epidemiological model), but is does not corresponds to rate at which susceptible individuals get infected (beta * (I_0+I_T)/N, in equation 4 that is not a fixed parameter but depends on all parameters via the prevalence (I_0+I_T)/N). We now explain this feature explicitly to avoid any confusion (page 10, line 247). For consistency with the literature in theoretical epidemiology, however, we still refer to beta as the 'transmission rate'.

Also, the birth rate and K have no effect on the proportion of cancers or selection, but surely these parameters are in reality somehow connected? This is just a thought as I had trouble with following the model, which surpassed my knowledge.

- The birth rate and the carrying capacity do not determine the equilibrium state that is eventually reached (including the proportion of neocancers, and the strength of selection

due to cancers), but they affect the dynamics of our epidemiological model before reaching the equilibrium (this is now written explicitly in page 11, lines 272-273). This is reminiscent to what can be found in other population dynamic models (e.g. the standard Lotka-Volterra competition equations where the baseline growth rate and the carrying capacity do not determine the outcome of competition).

Some other minor clarifications needed:
Line 330 -333, they claim “three cases of transmissible cancers have been identified in dogs, devils and bivalves”. There are more than three transmissible cancers, I think they should say transmissible cancers have been found on xx species or alter the text according to the number of transmissible cancers they are referring to.

- We rephrased this sentence as suggested (page 16, lines 357-359).

Lines 363 - 366. “More precisely, in Tasmanian devil, a new lineage of transmissible cancer has been shown to express the host's major histocompatibility complex class 1 (MHC1) molecules. … Simultaneously, however, an older lineage of transmissible cancers in Tasmanian devils avoids the immune response by downregulating the expression of the MHC1 molecules (Devil Facial Tumour 1;Caldwell and Siddle, 2017).”
In my opinion, DFT2 should not be defined as a new 'lineage' of transmissible cancer as implies that it evolved from DFTD, which is not the case. The manuscript first defines the devil cancer as DFTD, but then it uses DFT1 which is the same cancer. I know this might sound pedantic, but it would be good promote consistency in the devil literature because more and more papers are mixing the cancers and using them incorrectly. Ideally, they should explain the terminology early in the manuscript and keep consistency.

- We corrected these inconsistencies and made it clear that DFT1 and DFT2 are independent transmissible cancers in Tasmanian devil (page 16, line 358; pages 16-17, lines 390-394).

Else congratulation for this nice piece of work

- Thank you very much for these constructive criticisms.

Response to Reviewer #3

In their manuscript the authors probe the theory that the evolution of sexual reproduction in multicellular organisms was driven by the emergence and spread of contagious cancers. Intuitively these cancers are similar to parasitic infection and as such the authors propose the hypothesis that the emergence and spread of these cancers initiates fluctuations in selection to drive the evolution of sexual reproduction, increasing genetic recombination. The authors initially test this hypothesis by building a population genetic model with an assumption that transmissibility is determined by a single locus defining self/non-self. This model predicts that fluctuations in selection are not significant enough to drive the emergence of sexual reproduction.
The population model is then supplemented with an epidemiological model to link the proportion of 'neocancers' and the selection coefficient. They are then able to vary a number of parameters to determine which may produce the outcome of sexual reproduction. Again, the emergence to sex is unlikely in most scenarios, with the exception of where the transmission rate is high and selection is high.
The authors propose that the genetic link between newly emerged contagious cancers (so called neocancers) and their host is tight, which reduces selection and the 'Red Queen' scenario which would favour sexual reproduction.
The paper articulates an interesting hypothesis and builds the models logically to test this hypothesis. The primary issue with the models is their simplicity, numerous assumptions are made to simplify the model, such as the presence of a single locus determining self/non-self and an assumption of no mutation in tumour and host in the population model. These assumptions are obviously fundamental to the outcomes of the tests.

- We thank Reviewer #3 for this succinct and accurate summary of our study, and for this positive feedback.
- Making assumptions should not be considered as an “issue” but rather as a necessary step to formally test whether an hypothetical process makes sense. All models have limitations — the quest of a modeler is to seek a balance between tractability and the number of interesting phenomena that can be included. Of course, one can always relax more and more assumptions; one has to seek a balance between this and choose judiciously what is best left for future study. For instance, we implemented mutations (page 5, line 118) but we do not explicitly model any evolutionary process that occurs during the development of a tumour (page 6, lines 125-126) because including multi-level selection would have considerably reduced the tractability of our models. We hope that altogether the present analysis captures well the determinants of the Red Queen scenario in the case of infection by transmissible cancers and the robustness of the key finding.
- We agree, however, that it is absolutely crucial to discuss the limits of our models, and to what extent our conclusions hold with more complex features. In the revised version of our manuscript, we therefore relaxed some assumptions. In particular, we increased the number of genetic loci determining self/nonself recognition (as suggested by Reviewer #3; see below), we considered facultative sex as an ancestral trait, and we implemented vertical transmission of cancerous cells.

Central to this is the assumption that a single genetic locus determines self/non-self. Thus, the likelihood that the cancers will transmit to 'matched' individuals is very high and explains the genetic link between the host and cancer. The authors should introduce additional loci to the genetic model and test the result. This could be done in a stepwise manner to assess the impact on selection.

- In our new supplementary analyses, we increased the number of genetic loci underlying the interaction between multicellular organisms and transmissible cancers (Figure S3). Neoplasia dampens Red Queen dynamic, and this effect is even stronger when we increase the number of genetic loci (page 8, lines 196-199). Our results are therefore in line with previous studies showing that increasing the number of loci inhibits the evolution of

sex under the Red Queen hypothesis by reducing the strength of selection on each individual locus (Otto & Nuismer 2004, *Science* 304(5673):1018-1020) .

Similarly, in figure 1 a three locus model and one locus model is tested (Figure 1A). There needs to be a clearer explanation of these two models and why one is favoured.

- We could not solve analytically the three-locus model (i.e., two interaction loci + one locus controlling the reproduction mode), and therefore the link between the parameters and the Red Queen dynamics is inferred only on the basis of numerical simulations (Figure 1). Although a high proportion of neocancers seems to dampen Red Queen dynamics, one cannot be sure that this is the case over the entire parameter space. Solving analytically a simpler model with only one interaction locus gives us an inequality (Equations 2) confirming that neoplasia dampens the Red Queen dynamics. In this case, we know for sure that this effect occurs over the entire parameter space. An advantage of the three-locus model, then, is that it allows us to track explicitly the evolution of a modifier allele coding for sexual reproduction. The insights given by the two models are therefore complementary, rather than one offering more insight than the other.
- We hope that the explanation of the results is now clearer (page 8, lines 211-214).

In the methods (line 96) the authors state that host and cancer cells form two large populations of constant size. This needs to be clarified, does this mean that the cancer emergence is at a rate that results in equal cell numbers between host and cancers. This would not seem likely.

- The large, constant size of these populations is not meant to imply that they are the same size. These assumptions are made within each population, to allow us to ignore the effect of drift in each of them. We agree that our earlier writing was potentially misleading, and we therefore rephrased this sentence (page 5, lines 98-99).

Typo in line 150 - should read 'their sum is equal'.

- We corrected this typo.

Typo in line 170 - additional word - our

- We corrected this typo.
