# Supplementary material for: Transmissible cancers and the evolution of sex under the Red Queen hypothesis
Source: PLoS Biol. 2020 Nov 19;18(11):e3000916. doi: 10.1371/journal.pbio.3000916 (PMC7676742; doi:10.1371/journal.pbio.3000916)
Supplement: Attachment — Submitted filename: ResponseToReviewers.pdf [file pbio.3000916.s012.pdf]

Response Letter

Response to Reviewer #1 (signed: Joanna Masel)

My concerns have been satisfactorily addressed.

- We thank Prof. Joanna Masel for taking the time with this paper and for providing insightful comments throughout the review process.

One small requested change re equating the Red Queen or parasite coevolution hypothesis with fluctuating selection dynamics is that Haldane 1949 should not be cited in support of FSD. The FSD hypothesis began I believe with the Hamilton paper cited, and I don't think it is appropriate to back-project it onto earlier papers about host-parasite coevolution more generally.

- We agree that we should not have cited Haldane (1949) who does not refer to the implication of frequency dependence for the evolution of sexual reproduction.
- Although Hamilton (1980) provided the first theoretical investigation of the Red Queen hypothesis, several evolutionary biologists had by then suggested, more or less explicitly, that Red Queen dynamics may create preconditions for the evolution of sex/recombination in the host (Levin 1975, Hamilton 1975, Jaenike 1978, Bremermann 1980, Lloyd 1980). We therefore cite Levin 1975 and Hamilton 1975 who first suggested that coevolution with parasites may favour genetic mixing (page 2, lines 16).

Optionally on a linguistic note, I know it is common practice to equate "Red Queen" with FSD, but I do not believe it is appropriate given the far broader definition of Red Queen in the original van Valen paleontology paper, concerning extinction rates, that I believe first introduced the term Red Queen to evolutionary biology. Unfortunately, I think the effect of this linguistic practice is to help crowd out alternative and more realistic theories of host-parasite coevolution. This is a pet peeve of mine that is not at all specific to the manuscript at hand, which has been satisfactorily revised.

- The Red Queen theory originally had to do with the species-in-community level (Van Valen 1973). Nonetheless, the parasite avoidance theory for the evolution of sex was coined 'Red Queen' theory for the evolution of sex (Bell 1982), shortly after Hamilton's seminal paper (1980) got published. Hamilton himself commented in his autobiography « [a] notion of appellations controlées for our concepts have seemingly not yet developed in biology, so I am very happy to go along with the preferences of others who would call my present theme a "parasite Red Queen paper" » (*Narrow Roads of Gene Land, Volume 2: Evolution of Sex*, 2002, page 171). As a result, most papers investigating the implication of host-parasite coevolution for the evolution of sex refer the "Red Queen hypothesis" in their title or abstract (e.g., Howard & Lively 1994, Otto & Nuismer 2004, Salathé et al. 2009, Ashby & King 2015).
- Since us not using the term would not have any impact for its continued usage elsewhere (which is ongoing, see e.g. Koch et al. 2020 in J.Evol.Biol.'s September issue) and would simply make our paper harder to find for those interested in the topic, we chose to keep referring to 'Red Queen' theory. To avoid any confusion, however, we write "(not to be confused with the macroevolutionary Red Queen hypothesis; Van Valen 1973)" after defining "Red Queen hypothesis" (page 2, lines 16-17).

Response to Reviewer #2 (signed: Frederic Thomas)

This version is really much improved, I love it. However, I still have two comments (the second one being really important in my opinion):

- We thank Dr. Frederic Thomas for his very positive feedback and for his insightful suggestions during the review process.

1- I think there is a relevant paper that should be mentioned in your discussion.
Khosrotehrani, K. et al. (2005) Natural history of fetal cell microchimerism during and following murine pregnancy. Journal of Reproductive Immunology DOI: 10.1016/j.jri.2005.02.001
This paper in fact simulates our scenario using syngenic mice. Indeed, using syngenic mice these authors mimicked an asexual reproduction in mice, and they showed that when embryos are identical to their mother, microchimerism is significantly enhanced. They are not using cancerous cells, I agree, but this findings really supports our idea that vertical cell transmission is more difficult to control and/or to prevent when parent and offsprings are identical.

- We now refer to this paper after describing the implications of vertical transmission for the evolution of sex: "(as advocated by Thomas et al. 2019, and as suggested by the poor recognition of fetal cells during pregnancy in mice when embryos are identical to their mother, Khosrotehrani et al., 2005)" (page 14, lines 545-547).

2- I still think that there is a problem in the title. You wrote:
"In this paper, we investigate whether antagonistic interactions between hosts and transmissible cancers can promote the evolution of sex under the Red Queen hypothesis. We analyze a population genetic model of fluctuating selection and complement it with an epidemiological model. The latter model builds an explicit epidemiological setting that we then use to examine the likely parameter values that the population genetic model takes. This combined use of two models allows us to evaluate how likely it is for the modelled system to find itself within a selection regime where Red Queen dynamics can favour sexual reproduction."
The basic of the assumption for our paper was that transmissible cheater/cancer cells evolved at the same time as multicellularity. Due to their clonal reproduction the multicellular host cells could not recognise the transmissible cheater/cancer cells. Consequently, sexual reproduction evolved as an adaptive trait to prevent horizontal and/or vertical transmission of cheater/cancer cells. This was a one off evolutionary adaptation and hence once it appeared no further adaptations occurred. Importantly, our scenario should therefore not be regarded as a red-queen process, which is a constant, and ongoing battle between host and pathogens.

- In our research article, we decided to focus on the classical parasite avoidance theory for the evolution of sex that is based upon response to fluctuating selection (the so-called 'Red Queen hypothesis').
- We agree that the title we chose would not be a good fit if we were addressing absolutely all the points covered by Thomas et al. (2019), where the authors discuss the implication of transmissible cancers for the evolution of sex in a very broad evolutionary context. However, Thomas et al. 2019 do explicitly encourage others to study the topic in a Red Queen framework, and we indeed took this route. We also believe that referring to the "Red Queen hypothesis" in the title of our research article is necessary to put our study into the context of the existing literature on the implication of fluctuating selection for the evolution of sex (as highlighted in the abstract and the discussion). Indeed, in our model, we do consider "*a red-queen process, which is a constant, and ongoing battle between host and pathogens*".
